# Learning about causal relations that change over time: primacy and recency over long timeframes in causal judgments and memory

**DOI:** 10.1186/s41235-025-00614-9

**Published:** 2025-02-21

**Authors:** Benjamin M. Rottman, Yiwen Zhang

**Affiliations:** University of Pittsburgh, 546 Murdoch Building, 3420 Forbes Ave, Pittsburgh, PA 15260 USA

**Keywords:** Causal learning, Order effects, Serial position effect, Primacy, Recency

## Abstract

Being able to notice that a cause–effect relation is getting stronger or weaker is important for adapting to one’s environment and deciding how to use the cause in the future. We conducted an experiment in which participants learned about a cause–effect relation that either got stronger or weaker over time. The experiment was conducted with a typical procedure in which the learning cases were presented rapidly, and with a mobile phone procedure, in which participants experienced the cause–effect relation over 24 days. First, we found that people could detect the change in contingency. They were better at doing so in the artificial short timeframe task, but still could do so in the more realistic long timeframe task. Second, when making summary judgments about the cause–effect relation, participants exhibited a recency effect for most measures in the long timeframe, but did not exhibit a primacy or recency effect in the short timeframe. Third, though participants’ episodic memories for individual cause–effect events in the learning sequence were quite poor, they did exhibit primacy and recency effects in the short timeframe; these were attenuated in the long timeframe. These findings raise fundamental questions about causal learning; they suggest that people automatically recognize changes and store representations of the contingency during different phases of learning, but this ability is not predicted by most existing theories of causal learning.

**Supplementary Information:**

The online version contains supplementary material available at 10.1186/s41235-025-00614-9.

## Significance statement

This research was inspired by two aspects of real-world cause–effect learning. First, noticing a change in the strength of a cause–effect relation (e.g., a medicine starts to work better over time) is very important for making decisions (e.g., to continue to use the medicine or not), but has rarely been studied. We found that people are able to notice such a change. This finding challenges existing theories of causal learning, which do not predict this ability, and suggest that the theories need to be modified. Second, though order effects in causal learning have been well documented, a fundamental limitation in understanding how such effects apply in real-world situations is that they have been exclusively studied in artificial paradigms that last on the order of minutes. In real-world situations, however, people typically accumulate cause–effect experiences over multiple days or weeks. The current research tested whether transitioning from the short timeframe paradigm to a more realistic paradigm may lead to more of a primacy effect or more of a recency effect. This research also contributes to basic research particularly theories about learning over short vs. long timeframes, theories about the role of episodic memory for causal learning, and the meaning of order effects in causal judgments.

## Introduction

Many cause–effect relations change in strength over time. For example, many people feel tired when taking the allergy medication diphenhydramine (Benadryl), but become tolerant to the drug and no long feel tired after using it for multiple days (Richardson et al., 2002). Being able to notice that a cause–effect relation is changing over time would be useful; one could continue using a cause that is becoming more effective even if it was initially ineffective, or stop using one that is becoming less effective. Thus, one question of the current research is to assess whether people notice such changes.

A challenge to noticing a changing cause–effect relation in a real-world setting is that it requires comparing one’s recent experiences with experiences that occurred multiple days or weeks ago (e.g., experiences with Benadryl and fatigue during the last few days vs. a week or two ago). Learning that there is a change could be considerably easier in a standard causal-learning paradigm that occurs quickly and for which rehearsal can be used to keep items in working memory compared to a real-world learning sequence that stretches out across days and weeks and for which working memory is insufficient. To assess this question when participants learn in a realistic timeframe, we designed an experiment run over mobile phones to mimic how people learn over multiple days and weeks.

A second question that motivated this research is how learners make judgments about the cause–effect relation after experiencing a change in causal strength. Some prior research has found that participants’ judgments appear to focus on the initial learning episodes, a primacy effect, and other research has found that participants’ judgments appear to focus on the final learning episodes, a recency effect. Our participants answered multiple questions, such as whether they would continue to use the cause (e.g., continue to use Benadryl), how strong the overall strength of the cause–effect relation is across all the days, and other questions. Our smartphone study was designed to test whether learners exhibit a primacy or recency effect in a real-world long timeframe situation, and whether learners are able to flexibly use their memories of the experiences to answer different questions in different ways.

The third question that motivated this research was the nature of the relation between participants’ causal judgments and their episodic memories for individual cause–effect learning experiences. Whether people make causal inferences based on individual episodic memories of the learning experiences, or other types of memories that aggregate the experiences, is an open question. If the causal judgments and episodic memory findings are similar, it could suggest that when making causal inference people rely on the memories for individual items. If they are different, this suggests causal inferences work in a different way than simply aggregating stored memory traces of individual events.

In the rest of the introduction, we discuss these three questions sequentially.

### Question 1a: do people notice whether the strength of a causal is changing?

In many causal systems, the cause–effect relation does not always remain constant, but can become stronger or weaker over time. In living organisms, when a cause becomes stronger the process is called “sensitization,” and when it becomes weaker the process is called “tolerance” or “habituation.” Habituation and sensitization processes occur widely across many different domains in living organisms such as pharmacodynamics (Robinson, 2010; Stewart & Badiani, 1993), pain (Gold & Gebhart, 2010; Woolf, 2011), allergic responses (Husby, 2001; Lambrecht & Hammad, 2014), stress (Stroud et al., 2020), and psychological or behavioral responses of many sorts (Ardiel et al., 2017; Epstein et al., 2008; Rankin et al., 2009; Shen et al., 2020; Stewart et al., 1993). Processes with changing causal strength also occur in physical systems; they are often described as a component “wearing out” or “breaking in.” Brake pads are an example of both processes. Sometimes when brake pads squeak after being replaced, but through the process of driving and using the new breaks they stop squeaking. Additionally, as brake pads wear out they are designed to make noise to alert the owner that they need to be replaced. In sum, in many everyday situations the efficacy of a cause to produce an effect can change over time.

Being able to notice if a causal process is becoming stronger or weaker would allow an individual to adaptively respond to their environment. For example, if they notice that the causal process is changing they would be able to predict the future events in the sequence and take action to produce a desired outcome (e.g., increase the dose of a medication that is becoming less effective; replace the brake pads before they wear out completely).

However, there is little research on whether people notice changing cause–effect relations especially with binary variables (but see Rottman & Ahn, 2009). Though it may seem that noticing a changing cause–effect relation should be trivial, we contend that it is not trivial computationally, and use existing theories of causal inference to explain why. There are three main families of cognitive processes that could underlie causal learning; for a review of research on different accounts of causal learning, see Pineño and Miller (2007).

One process is **reinforcement learning (RL)** or associative learning (e.g., Rescorla & Wagner, 1972; Sutton & Barto, 1990), which involves sequentially updating a belief about the strength of the cause–effect relation. RL processes have long been proposed as models of human causal learning (e.g., Pineño & Miller, 2007; Shanks & Dickinson, 1988; Shanks et al., 1989, 1996). Standard model-free RL models assume that learners sequentially update a memory of the cue values and do not keep a memory of the historical record of the cue values. Not keeping a historical record of the cue values, nor memories of the learning experiences themselves, makes RL very efficient with regard to memory resources, but one implication is that standard RL algorithms can only report the current estimate of the strength of the relation, not whether the strength of the relation has changed. More sophisticated RL models that infer latent states (e.g., Courville et al., 2006; Daw et al., 2008) could potentially be used to detect different periods in the learning data, which could be used to infer a change in strength, and however, this ability is not part of standard RL models.

Another process that has often been proposed for causal learning involves storing memories of four **tallies**; the number of times the cause was present vs. absent and effect was present vs. absent (e.g., Cheng, 1997; Griffiths & Tenenbaum, 2005; Hattori & Oaksford, 2007). This class of theories is sometimes called “rule-based” or “statistical theories.” Storing tallies of the sequence of events does not allow a learner to notice that the causal strength is increasing or decreasing. Extensions of this theory could be proposed such that a learner could be looking for changes and if they detect a change, start to keep track of separate tallies for the two periods. However, such an extension would need to be fleshed out to explain how a change is noticed and when the change is believed to have occurred. And if a change is noticed, the learner would need to keep track of 8 tallies instead of 4, which would presumably be challenging.

A third process involves using **contextually bound episodic memory**. Episodic memory has not been discussed much within the causal learning literature; however, some theories of decision making in reinforcement learning have proposed that people make decisions by sampling from episodic memory (e.g., Bornstein et al., 2017; Gershman & Daw, 2017; Wang et al., 2022; Wimmer & Büchel, 2016). In the current research, we use the term “contextually bound episodic memory” to mean that the learner stores memories of individual experiences with the cause and effect, and that these memories are bound to contextual features such as *when* the event occurred and/or other stimuli occurring at the same time. (Another possibility is that even if our participants did not have strongly contextually bound episodic memories, that they may have still had non-contextual episodic memories of the cause and effect and used these for making causal judgments. However, this version of episodic memories is almost indistinguishable from storing tallies.)

If a learner has memories of individual cause–effect events and can remember when the event occurred, then it would be possible to retrospectively recall events from earlier in the learning sequence and compare them to events later in the learning sequence, and notice a change the strength of the cause–effect relation. Of course, exactly how this occurs would need to be fleshed out; it could involve calculating tallies from memories of the earlier events and later events and then comparing the tallies, which as explained above would be challenging. But the point is that contextually bound episodic memory could in theory allow for noticing a change in strength.

In summary, out of all three standard theories, only contextually bound episodic memory is sufficient to support noticing a change in causal strength. The predictions for these three theories are summarized in the top row of Table 1.

**Table 1:** Predictions of each cognitive process for the three main questions

Questions	Episodic memory	Tallies of events	Reinforcement learning
Q1. Aware of change	Possible, but impacted by which items can be retrieved	Chance performance	Chance performance
Q2. Summary measures of cause–effect relation	Impacted by which items can be retrieved and by question interpretation	Average of all learning episodes	Recency
Q3. Episodic memory	Impacted by which items can be retrieved	Chance performance	Chance performance

The broader point is that standard theories of causal learning either cannot handle such an inference, or such an inference would be challenging. At the same time, we contend that cause–effect relationships with changing strengths are common in everyday life, and that being able to notice the changes would be useful. Thus, one goal of the current research is to assess whether people are able to notice such changes.

### Question 1b: is there a difference in the ability to notice that the strength of a causal relation is changing across short vs. long timeframes?

Most studies of causal learning involve presenting a set of learning events to participants rapidly. However, we contend that in real-life situations, causal learning often occurs over days or weeks, not minutes (e.g., learning whether a new medicine you are trying is working, learning how to interact with a new colleague, learning whether you are sensitive to gluten, learning how to improve your sleep). We have recently conducted a series of causal learning studies in which the learning events are spread out over many days, to assess whether causal learning abilities found in the typical rapid short timeframe setting are similar to those in a more realistic long timeframe setting (Willett & Rottman, 2021; Zhang & Rottman, 2023a, 2023b).

We believe that it is crucial to study whether or not people notice changes in causal relationships in a more realistic long timeframe setting. The reason is that when the learning events are spaced out over many days, noticing a change involves some sort comparison between events that occurred many days ago and events that occurred within the last few days, or at least a mental summary of events that occurred many days ago vs. a summary of events that occurred recently. To the extent that it is difficult to recall the events or summaries of events that occurred many days ago, noticing a change would be challenging.

To be specific, consider the proposed extension of the tallies theory in which a learner keeps two sets of four tallies. Keeping track of eight tallies might be somewhat possible in a short timeframe setting in which rehearsal could be used to keep track of tallies in working memory; however, in a long timeframe setting in which only long-term memory is used, keeping track of eight tallies may not be feasible. In the current study, we tested whether participants were aware of the change in causal strength in both a short timeframe and a long timeframe condition.

### Question 2a: are there order effects in causal judgments?

Order effects in summary judgments have been studied in many fields including conditioning (Pineño & Miller, 2005), statistical learning (Bulgarelli & Weiss, 2016), probability estimation (Peterson & DuCharme, 1967), juror decision making (Furnham, 1986), persuasion (Miller & Campbell, 1959), stereotype formation (Johnston, 1996), and implicit biases (Fourakis & Cone, 2020). For a well-known example of an order effect in a summary judgment, people tend to judge individuals more positively if they are initially described as having positive personality traits, despite subsequent negative traits, relative to the reverse (Anderson & Jacobson, 1965; Asch, 1946; Sullivan, 2019). This is evidence of a *primacy effect*, where initially encountered information has a stronger influence on the final judgment than more recently encountered information. Conversely, a *recency effect* occurs when more recent information has a stronger influence on the final judgment.

Within the field of causal learning, the focus of this research, order effects have typically been studied by having participants experience a positive contingency between a cause and effect during the first half of the learning experience, and then a negative contingency during the second half (or vice versa), so that the average is zero. If participants’ final judgments are positive, this is considered to be evidence for a primacy effect—that they weighted the initial experience more—and if it is negative it is considered to be evidence for a recency effect. It is important to note how this one paradigm, in which the experiences change from the beginning to end of learning, can be used to study both “order effects” (Q2) and whether participants are aware of a change in contingency (Q1); the difference is just whether participants are asked to make a summary judgment at the end of learning or to reflect on whether the causal strength has changed.

Some research on order effects in causal learning has found primacy effects (e.g., Collins & Shanks, 2002; Curley et al., 1988; Danks & Schwartz, 2005, 2006; Dennis & Ahn, 2001; Glautier, 2008; Marsh & Ahn, 2006; Matute et al., 2002; Yates & Curley, 1986). Other research has found recency (e.g., Catena et al., 1998; Collins & Shanks, 2002; Glautier, 2008; López et al., 1998; Vadillo et al., 2004). These primacy and recency effects in causal learning have typically been subtle; participants’ judgments are somewhat more similar to the experiences in the first half or the second half of the learning phase, but not dramatically which implies that it is unlikely that participants entirely ignore or forget the other half.

The three theories of causal inference previously mentioned also make different predictions about order effects; see the middle row of Table 1.^1^ Standard RL models predict recency effects (e.g., Dennis & Ahn, 2001; Wasserman et al., 1996). RL models sequentially update the estimate of the strength of the relation between the cause and effect. The sequential updating means that at any given time the estimate is impacted more by the most recent evidence. In fact, recency effects can be considered to be adaptive. In a changing world, it is reasonable to assume that recent events are more predictive of the future than are events from the distant past (Bouton, 1997; Courville, 2006; Courville et al., 2006; Daw et al., 2008; Gershman et al., 2010 for related work from a rational perspective).

In contrast, the standard Tally theories assume that people develop a tally for the entire learning sequence and then use the tallies for judging causal strength. Because tally theories treat events at the beginning vs. end of learning equally, the judgments reflect an average over all learning experiences.

Contextually bound episodic memory could lead to a wide variety of different outcomes due to two processes at play: a learner’s ability to recall individual events and a learner’s decisions about how to use the events that they recall to answer the questions. First, consider which events a learner recalls. Suppose that when making a summary judgment about a cause–effect relation that a learner first recalls individual events and then uses these events for making the judgment. If they tend to recall more of the initial events, the summary judgment would exhibit a primacy effect, and if they tend to recall more of the later events, the summary judgment would exhibit a recency effect. Of course, in many memory paradigms participants are more likely to recall the first few items and the last few items than the middle items (see, for example, Brown et al., 2007). If these two effects balanced out, then the summary judgment would reflect an averaging of the two halves, but if there is an imbalance it would tip the summary judgment toward primacy or recency. There are many reasons in memory paradigms that could lead to stronger primacy or recency; some of these are discussed in the general discussion.

Contextually bound episodic memory could also allow a learner to flexibly answer different summary questions about the cause–effect relation in different ways depending on how the learner interprets each question. In particular, some questions may be interpreted to be asking about the entire learning experience, whereas others may prompt a learner to focus more on recent events. In fact, in Matute et al.’s (2002) study, when participants were asked to judge the strength of the cause–effect relation, they roughly averaged across all the data, but when making predictions of the effect they exhibited recency. Our study sought to replicate and extend Matute et al.’s finding with additional dependent measures. We asked multiple different summary measures. For example, in the ‘Average Strength’ question, we specifically asked participants to consider all 24 events. If they answer this question differently from the other questions (e.g., a causal strength measure that does not specifically tell participants to focus on all the experiences), it is a sign that they are choosing to weigh earlier or later questions differently for the other questions. In the methods section, we provide more details of why we asked each question.

In contrast to contextually bound episodic memory, the Tally and RL theories predict that all questions at would be answered the same way, not differently impacted by primacy or recency. For Tallies, question wordings could impact how participants weigh the four tallies (Buehner et al., 2003), but since the tallies do not record which events occurred when, question wordings could not impact primacy vs. recency. For RL, since the leaner only has access to the final cue value, this is all they can use to make any judgment.

In sum, assessing whether participants are aware of a change in causal strength (Question 1) as well whether there are order effects in participants’ summary judgments (Question 2) provides insight into how detailed participants’ memories are and how they may use the memories in flexible and sophisticated ways for thinking about a causal relation.

### Question 2b: is there a difference in order effects across short vs. long timeframes?

All the prior research on order effects in causal inference has used short timeframe studies. One goal for the current research was to assess whether order effects in causal judgments are impacted by the timeframe of learning. There are many potential reasons for different outcomes. Some of these reasons connect directly to the theories of causal inference already discussed, RL, Tallies, and Contextually-Bound Episodic Memory, and some of the reasons connect to other theories and findings from other areas of psychology.

#### Shift toward recency in the long timeframe

One hypothesis is that there will be a shift toward recency when learning over a long timeframe. A couple pieces of evidence in short timeframe learning implicate WM as responsible for primacy effects and RL as responsible for recency effects in causal judgments. First, individual differences in WM are correlated with stronger primacy effects in causal judgments (though the correlation is weak). Second, increasing WM load leads to more recency effects in causal judgments (Luhmann & Ahn, 2011; Marsh & Ahn, 2006). The proposed explanation for these findings is that primacy effects are believed to be due to forming a strong initial causal hypothesis, supported by WM; when WM is taxed too much then the RL system takes over, and RL produces recency effects.

With regard to long timeframe learning, learning in the long timeframe can be viewed as more complex since the learning task is embedded within all the other events of daily life, and furthermore, WM cannot support learning that takes place over this timescale. In contrast, the RL system is believed to be highly robust and available even under cognitively challenging situations (e.g., Collins, 2018; Collins & Frank, 2012), so should be able to support learning over long timeframes. Thus, it is plausible that we would see a shift toward recency in the long timeframe.

Zhang and Rottman (2023b) also found some hints of a shift toward a recency effect in long compared to short timeframe causal learning. In that study, participants had good memories for all the events—the shift toward recency likely reflected *using* the more recent events for making the judgment. However, that study was not specifically designed to test for order effects and used a different paradigm than the one studied here in which the contingency changes, so it is not clear that the findings will translate to the current case.

Additionally, a few studies have found recency effects in participants’ memories accumulated over long timeframes over the course of their everyday lives. Baddeley and Hitch (1977) found recency effects in participants’ recall of rugby games, and Pinto and Baddeley (1991) found recency effects in participants’ recall of where they parked their car each day over the prior 12 working days. Note, however, that these studies are quite different from typical studies on order effects; they do not test all the way back to the participant’s first experience and they are not experimentally controlled.

#### No differences between the short and long timeframes

A second hypothesis is that there may be no differences between the short vs. long conditions. Indeed, our prior study investigating long timeframe learning did not find a difference (Willett & Rottman, 2021).

Furthermore, there are influential theories in both literature on animal learning (Gallistel & Gibbon, 2000) and episodic memory (Bjork & Whitten, 1974; Brown et al., 2007; Crowder, 1976, 1993; Howard & Kahana, 2002; Nairne et al., 1997; Tan & Ward, 2000) that are ‘timescale invariant’; these theories predict that if all of the timings of the events of the study are proportionally expanded, there should be no impact on learning or memory. The causal learning and causal judgment portions of this study were stretched out roughly proportionally (see Online Appendix 1 for more details about the timing in the memory judgments).

Another piece of evidence comes from a unique study on an individual’s memory for details about opera performances over 25 seasons (Sehulster, 1989). This study found both primacy and recency effects in memory for individual items. Though there is no direct comparison to the short timeframe, the main conclusion was that the item order effects in this long timeframe study were quite similar to those often found in short timeframe studies.

#### Shift away from recency in the long timeframe

A third hypothesis is that there will be a shift away from recency in the long timeframe compared to the short. In causal judgments, such a shift would appear as any movement from recency toward primacy; this does not require a finding of recency in the short and primacy in the long, just any movement away from recency and toward primacy.^2^

This hypothesis can be motivated by the theory that episodic memory supports causal inference. If when making a causal judgment a learner uses memories for specific events in the learning series, and if they are more likely to recall earlier events compared to later events in the long timeframe compared to short, this would lead to a shift away from recency and toward primacy. Likewise, if in the short timeframe they mainly recall later events, a recency effect, and in the long timeframe their recall for all events is quite poor so the recency effect goes away, their causal judgment would also reflect a shift away from recency.

In fact, Mack et al., (2017) found evidence for a shift away from recency in a study on memory for word lists. Inspired by the fact that almost all experimentally controlled research on item order effects has used interstimulus intervals on the order of seconds, Mack et al., (2017) conducted three smartphone studies in which participants were shown word lists, one word each hour over the course of a day, and recalled the list at the end of the day, for many days in a row. When participants recalled the words at the end of the day, there were significant primacy effects but smaller and non-significant recency effects. Mack et al. argued that their long timeframe findings exhibited considerably smaller primacy and recency effects compared to short timeframe studies, though there was a remaining primacy effect and no remaining recency effect in the long timeframe. If a similar process occurs for memories of a sequence of causal events, and if people use the recalled memories of individual events for making a causal judgment, this would predict a shift away from recency in the long timeframe compared to the short.

#### Relation with ability to notice a change

One other factor at play is whether participants are equally able to notice a change in contingency in the long timeframe as in the short (Question 1b). If participants are able to notice the change in the short timeframe, but not the long timeframe, or to a lesser degree in the long timeframe, this could impact how participants answer the different summary questions. For example, if they are able to notice the change in the short timeframe, they may give somewhat different answers to the four summary measures in the short timeframe, and if they are less able to notice the change in the long timeframe, they would give similar answers to the four questions in the long timeframe.

In sum, there are many potential reasons for shifts in order effects in a short vs. long timeframe. Thus, a goal for this study was to assess whether in fact there are differences or not.

### Question 3: are the order effects in causal judgments similar or different to item order effects in episodic memory, and across the short and long timeframes?

As already explained, contextually bound episodic memory is one of the processes that could support causal learning. However, there is little research on whether people use contextually bound episodic memories for causal learning and judgment. In the current study, we compared participants’ causal judgments to their memories for individual cause–effect episodes; if episodic memory is used for making causal inferences, then any item order effects should be similar across the two.

To study if learners use episodic memory for making causal inferences, in our study each learning episode included a contextual image in addition to the cause and effect. At the end of the study, after asking participants’ causal judgments, we showed participants the contextual images and asked them to recall *when* the image occurred, which is typically called ‘temporal order memory’, and asked them to recall whether the cause and effect were each present or absent during that learning episode.

One challenge of comparing causal judgments and episodic memories is that causal judgments can only exhibit a primacy effect, a recency effect, or neither, but episodic memory can reveal both primacy and recency simultaneously. In memory studies like cued and free recall of learned lists as well as order reconstruction tasks (e.g., Ebbinghaus, 1913; see Brown et al., 2007, for a review), people tend to have better recall for items presented at the beginning and end of a sequence versus items presented in the middle of a sequence, though there are many factors that can moderate the strength of the primacy effect and the recency effect; some of these are mentioned in the general discussion.

In contrast, in causal learning studies (and in other paradigms in which participants make one summary judgment at the end of the learning task), it is not possible to observe both primacy and recency. In causal learning studies, the typical paradigm randomizes participants to experience a positive contingency for the first half of items, and a negative contingency for the second half of items (or vice versa). In the positive-then-negative order, a positive judgment would be considered a primacy effect, and a negative judgment would be considered a recency effect. However, suppose that the final judgment is negative (a recency effect), it is still possible that the participant also has an especially strong memory for the first few experiences, just that their memory for the most recent experiences is even stronger. In summary, order effects in causal judgments can only reveal a general tendency toward weighting the first half or the second half of the learning experiences more.

In sum, our study tested whether order effects play out in similar or different ways in causal judgments and in episodic memories. If they are different, this suggests that they rely on different processes. If the findings are similar, this could suggest that causal inferences rely on memories for individual events (or that there are two separate processes but that they work similarly). The bottom row of Table 1 shows the predictions that the three theories make when episodic memories are queried; Tallies and RL predict chance performance as they do not store episodic memories. By comparing the results of the summary measures and the episodic memories, it can elucidate whether similar or different processes are being used. We also compared causal judgments and memories across short vs. long timeframes, because the processes that drive causal learning may differ across timeframes (Question 2b).

### Current study

In the current experiment participants learned about cause–effect relations that either got stronger or weaker over 24 events. There were two conditions. In the ‘short timeframe’ condition, the events were presented back-to-back in the standard paradigm. In the ‘long timeframe’ condition, one event was presented each day for 24 days. We assessed three types of measures: (1) whether participants noticed the change in contingency, (2) multiple different judgments about the cause–effect relation, and (3) episodic memory.

## Methods

### Participants

Two hundred and twenty-three participants enrolled in the study. In our preregistration, our goal was to have at least 50 participants in each of the four main conditions. Participants were eligible for the study if they were fluent in English, between 18 and 30 years old, owned a smartphone, intended to complete the entire study, and had not participated in a similar study with our lab. The participants were recruited from the community near the University of Pittsburgh, and most of the participants were undergraduate students but not all. Participants who successfully completed the entire study were paid $30. Our final analyses included 186 participants after excluding 6 who missed more than three days of the study, 7 who counted on their fingers in the short task, 7 who wrote down data in the long task, and 17 due to a programming error. Their average age was 20.1 years (*SD* = 4.85), and 97% were under 30 years.

### Datasets and design

**Fig. 1:**
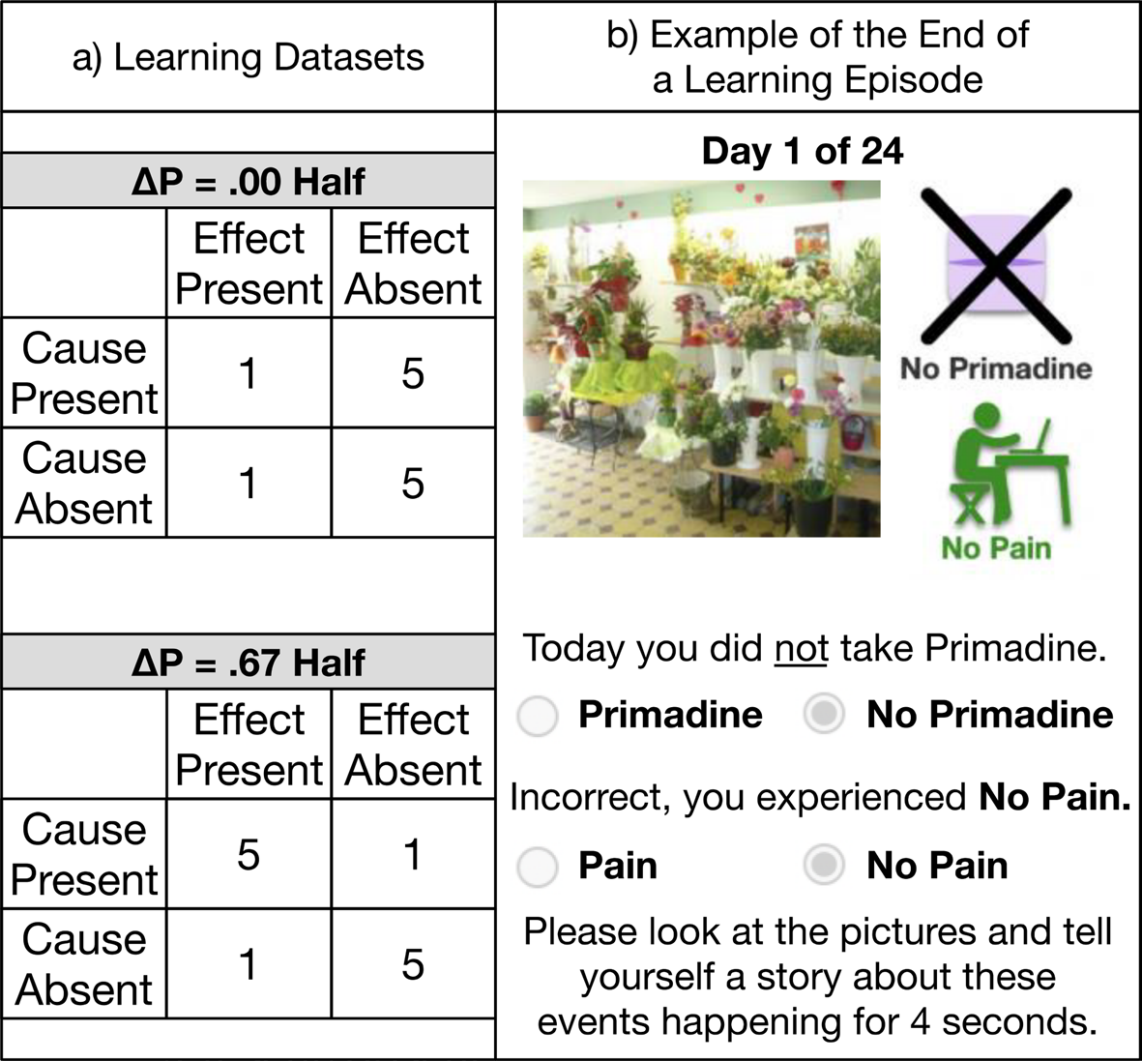
**a** Learning datasets for the two halves. **b** Example of the end of a learning episode

Participants observed 24 episodes in which they learned about the relationship between a binary cause and effect (Fig. 1a).^3^ Across the entire dataset, the cause–effect relation was moderately positive. ΔP is the difference in probability of the effect occurring when the cause is present vs. absent: *p*(*e* = 1|*c* = 1) − *p*(*e* = 1|*c* = 0). Power PC (Cheng, 1997) is a metric designed to capture causal strength and normalizes ΔP on the base rate of the effect such that if the effect is always absent when the cause is absent they are the same: Δ*P*/*p*(*e* = 0 *| c* = 0). Both of these metrics were moderately positive: Δ*P* = 0.33, Power PC = 0.40.

The relation either increased or decreased in strength such that it was nonexistent in one half of events (Δ*P* = 0, Power PC = 0) and strongly positive in the other half of events (Δ*P* = 0.67, Power PC = 0.80). In both halves, when the cause was absent, the effect occurred 1 out of 6 times. From the perspective of a causal system in which both the target cause and background causes can generate the effect (e.g., Cheng, 1997; Griffiths & Tenenbaum, 2005), holding this constant means that background factors that can produce the outcome do not change. The only thing that changes across the two halves is the strength of the cause–effect relation.

Participants were randomly assigned to either the increasing (*n* = 90) or decreasing (*n* = 96) conditions, and they saw a short and a long version of the same dataset. In the short timeframe, they made judgments after observing 24 episodes back-to-back, presented just seconds apart. In the long timeframe, they made judgments after observing one episode per day for 24 days. The order of tasks was counterbalanced; half of participants completed the short task before (*n* = 92) and half after (*n* = 94) the long task.

### Procedure

#### General procedure

Participants used their personal smartphones to complete the entire study (both the short and long timeframe conditions). The experimental website was created using our PsychCloud.org framework (Rottman, 2019). The entire study lasted 29 days. On Day 1, participants completed a short 8-event introduction task in our laboratory. Additionally, participants in the short task-first condition then completed the short task in its entirety in the laboratory on their phones.

On Days 5–28, participants completed Events 1–24 of the long task.^4^ On Day 29, participants returned to the laboratory to do the testing phase of the long task, complete the short task on their phones if they were in the long task-first condition, and receive payment. Due to the onset of the COVID-19 pandemic, a portion of participants completed the last day remotely via video conference calls.

Participants received automated text-message reminders to complete the task each day at 10 am, 3 pm, and 8 pm. They were told that if they missed more than three days of the study, the study would be terminated. If participants missed one (11%) or two (3%) days of the study, the subsequent episodes were pushed back so that they experienced each on a different day. Most participants returned to the laboratory one day after completing Event 24 (88%), but some returned on the same day (10%), two days later (2%), or three days later (< 1%), depending on the number of missed days and their availability.

In the short timeframe, the learning events occurred one after another, about 15 s apart on average. Right at the end of the 24 learning events the summary causal judgments were made, and then, participants answered a series of memory test questions. In the long timeframe, the learning events occurred once per day, and the testing phase (causal judgments and memory probes) usually occurred one day after the last learning phase. Details about the actual interpresentation intervals and retention intervals that participants experienced appear in Online Appendix 1.

#### Cover story

In each task, participants learned about the relationship between a fictitious medicine (cause) and a possible side effect. The medicine was either called “Primadine” or “Garatam.” The side effect was either arthritis pain or dizziness; participants were randomly assigned to one of these medicine names and one of the side effects for the short timeframe condition and the other for the long. In the manuscript, we use Primadine and pain exclusively, but the correct medicine and side effect were shown to participants given their random assignment. Participants were told the following cover story:

“Please imagine that due to a health condition, you are on a medication called Primadine. In addition to that health condition, you also sometimes have pain from arthritis. You have heard that sometimes Primadine can improve or worsen pain as a side effect. Some medications happen to improve arthritis pain as a side effect by decreasing the autoimmune processes that cause inflammation and pain in arthritis. Some medications happen to worsen arthritis pain as a side effect by increasing the autoimmune processes that cause inflammation and pain in arthritis.”

#### Within an episode

Participants experienced the same sequence of events in a learning episode for both the short and long conditions. First, participants saw an image and were told to imagine that this was a scene from that day in their life. The collection of images we used was designed so that each image was a unique setting (e.g., flower shop, gym, dentist office), which was found to scaffold episodic memory (Robin & Olsen, 2019). The image was displayed by itself for three seconds, and its purpose was to create a distinct context for each episode, so that we could test participants’ episodic memories after the task. Second, an icon and text appeared to indicate whether the participant did or did not take the medicine that day. Third, to increase task engagement and ensure that they processed this information, participants verified the cause as present or absent by pressing a radio button. Fourth, participants predicted whether they would or would not experience the side effect. Fifth, participants were given feedback and told the true state of the effect for that episode. Sixth, they pressed a radio button to verify the state of the effect. Seventh, participants were told to look at the pictures and tell themselves a story about the events for four seconds (Fig. 1b). After four seconds, a submit button appeared that they pressed to complete the episode. In the short task, participants would immediately move on to the next episode. In the long task, they were logged out of the website and completed the next episode on the following day. At all points during the study participants were unable to go back and see what happened on a prior episode.

### Event-by-event predictive strength during learning

During each learning episode, participants learned whether the cause was present or absent and then predicted whether the effect would be present or absent. We converted these into a measure of their beliefs about the strength of the relation between the cause and effect using the ΔP formula (Allan, 1980): *p*(predicted effect present | cause present) − *p*(predicted effect present | cause absent). This equation was run once for each participant for Events 7–12. Doing so turns these six predictions into one number between -1 and 1 that reflects whether a participant expects that the effect will be more likely when the cause is present vs. absent.

We calculated event-by-event predictive strength for the second quarter of events, Events 7–12, to test whether participants had successfully learned the contingency.^5^ If so, participants in the decreasing condition should have a higher predictive strength than the increasing condition, because in the decreasing condition the contingency starts out high and in the increasing condition it starts out low. This measure also provides a test of whether acquisition is stronger in the short vs. long timeframe.

### Summary measures and awareness of change

After the 24 learning episodes, six different dependent measures were collected. Each question was asked on a separate page, and after moving on participants could not go back and change their answers. Below, we explain and discuss each measure in the order that the questions were asked.^6^ With the exception of Awareness of Change, we call all of these “summary measures,” as they either use a single question to judge the cause–effect relation as a whole, or use multiple questions that are combined into a single measure of the cause–effect relation.

Each of the measures on the − 7 to + 7 range was transformed into the range -1 to 1 for data analysis. On average, the medication was a moderate cause of the side effect and “worsens” the pain. For ease of interpretation, we reverse-coded the measures so that participants’ answers should be positive. After these transformations, a judgment of + 1 means that the medicine causes the side effect and strongly worsens pain, and a judgment of − 1 means that it inhibits the side effect and strongly improves pain.

#### Causal strength

At the end of the learning phase, participants made a causal strength judgment “Do you think that Primadine worsens or improves pain?” on a scale of − 7 (“strongly worsens”), 0 (“no influence”), to + 7 (“strongly improves”). A causal strength judgment like this is the most typical dependent measure in the causal-learning literature.

#### Final predictive strength

Similar to Matute et al. (2002), participants answered two questions involving future predictions: “Imagine that ‘tomorrow’ (Day 25), you [take/do not take] Primadine. On a scale of 0–100%, what do you think is the likelihood that you would experience pain?” We used the Δ*P* formula to transform their responses into a measure of final predictive strength: *p*(effect = present | cause = present) − *p*(effect = present | cause = absent).

#### Continue use

Participants answered “Do you think you should continue to use Primadine?” (− 7 = “definitely no”, 0 = “unsure”, + 7 = “definitely yes”). Unlike some of the other questions that are more abstract and may be difficult to answer if a participant has noticed a change in contingency (e.g., causal strength), this question about intended behavior is more concrete and a change in contingency could inform how to respond (e.g., if it has started to work better, perhaps it makes most sense to continue to use it). After the transformation, judgment of + 1 means that participants wanted to stop using the medicine, and a judgment of − 1 means that participants wanted to continue using it.

#### Awareness of change

To assess if they were aware of the change in contingency from the beginning to the end, participants answered two causal strength questions: “Think about when you [first started/were more recently] using Primadine, did Primadine worsen or improve pain?” (− 7 = *strongly worsens*, 0 = *no influence*, + 7 = *strongly improves*). If the judgment for the end is higher (lower) than the beginning, participants were aware that there was an increasing (decreasing) trend in causal strength.

#### Average strength

Participants then answered, “On average over all 24 days do you think that using Primadine worsens or improves pain?” (− 7 = *strongly worsens*, 0 = *no influence*, + 7 = *strongly improves*). Since this question came right after the awareness of change questions in which participants were asked to focus on the beginning vs. end, and since it mentions ‘all 24 days’, it should have been clear that the goal of this question was to think about the whole set of experiences.

This question is important in comparison with the Causal Strength question. The “Causal Strength” question, which is most common in the field, is vague in that it does not direct the learner to focus on a particular set of experiences. Some participants might interpret the question as asking more about the initial experiences, whereas others might interpret it as asking about the more recent experiences—this could lead to order effects that are merely due to question interpretation not which experiences can be recalled. Additionally, if the causal strength questions is asked repeatedly, some participants might interpret it pragmatically to mean what is the causal strength since the question was last asked (e.g., Hogarth & Einhorn, 1992; Vadillo et al., 2004).

By examining the responses to the causal strength question and the average strength question, we can get a better understanding of reasons for order effects. If people are not aware of a change, then presumably their answers will be the same for the average strength question and the standard causal strength question. However, if they are aware of a change, then it is possible that these questions will diverge if people interpret the standard causal strength question as really asking more about the recent experiences or more about the initial experiences. They might also be aware of the change, but still answer these questions similarly; this would suggest that when making a causal strength judgment they are considering all the experiences.

#### Tally strength

Participants recalled the number of times they experienced each of the four event types (cause = present/absent, effect = present/absent): “Of the 24 days in the study, how many days did you see a picture in which you [did/did not] take Primadine and [did/did not] experience pain?”. We combined these four tallies into one measure of strength using the Δ*P* formula (Allan, 1980): *p*(remembered effect present | cause present) − *p*(remembered effect present | cause absent). The order of the four event types was randomized for each participant and each question was presented on a different page. Participants were able to enter any number from 0 to 24, had to verify their answer, and then could not go back to change any previous answers.

This sort of measure has been used in studies of causal learning and inference at least as far back as Arkes and Harkness (1983). There were two main reasons for asking the Tally Strength measure. First, this measure is directly related to the Tally theories that assume that a learner is keeping track of these four tallies and uses them to make judgments. Second, it provides an especially objective assessment of participants’ learning about the overall contingency between the cause and effect that should not be subject to the sorts of interpretations that could lead to primacy or recency effects for the other measures. If this measure reveals an order effect, it is most likely due to the learning process rather than the judgment process. Comparing this measure to the other summary measures can help reveal reasons for order effect and underlying cognitive processes that fit with the patterns.

### Memory measures

After the summary measures, participants’ memories were probed in two ways: contextually bound episodic memory and temporal order memory. These two are often studied in the literature on episodic memory and get at different aspects of episodic memory (whether the entire event is contextually bound vs. when it occurred; see for example Brown et al., 2007, for a discussion). Participants were shown each contextual image from the learning task in a random order, and answered the two memory questions, and then pressed a submit button to move on to the next image.

To assess if participants had **contextually bound episodic memories** of the events, for each image, they had to recreate the event from memory by indicating whether the cause and effect were each present or absent. For each image, we coded participants’ episodic memories as accurate if they correctly recalled the state of both the cause and the effect from the learning phase, so chance performance was 0.25. For simplicity, we sometimes call this measure just “episodic memory”, though temporal order memory is also a type of episodic memory. Contextually bound episodic memory is particularly relevant to learning the cause–effect relation in that it involves memories of specific episodes of the cause and effect.

To assess **temporal order memory**, for each image participants typed in a number between 1 and 24 to answer the question “On which day (out of 24) did you see this scene?” The way we assessed temporal order memory only involves the contextual image, not the cause and effect, so temporal order memory is less relevant to the cause–effect relation. Still as temporal order memory often exhibits item order effects, it provides useful context for all the measures that are about the cause–effect relation.

## Results

The study design and analysis plan were registered after data collection began but before looking at the data; see https://osf.io/px4tb/ for the analysis plan, data, and analysis code. The data were analyzed with the ez package (Lawrence, 2016) for ANOVA’s, effectsize package (Ben-Shachar et al., 2020) for η_p_^2^ and Cohen’s *d*, and the parameters package (Lüdecke et al., 2020) for confidence intervals. We calculated *p* values for all analyses and used *ɑ* = 0.05. We calculated Bayes Factors (BFs) using the BayesFactor package (Morey & Rouder, 2018) for all analyses except for the random effects regressions, for which BFs are hard to calculate with standard packages. For the Bayesian tests, we use the default two-sided ‘JZS’ prior in this package, which is described by Rouder et al. (2009), Rouder and Morey (2012), and Rouder et al. (2012).

Each participant completed a short and a long version of the task, so experiencing the first task could potentially have a carryover or contrast effect on the second task. To test this, we conducted a 2 contingency change (increasing vs. decreasing, between subjects) × 2 task length (short vs. long, within subjects) × 2 task order (first vs. second, between subjects) ANOVA for each dependent measure. None of the two-way or three-way interactions were significant, and all of the BFs were less than 1 (in the direction of supporting the null hypothesis of no difference); most were in the range of 3:1 to 5:1 in support of the null. Because there were no significant interactions, we report within-subject analyses in the main paper. However, out of an abundance of caution we report between-subjects analyses using only the first task that participants completed in Online Appendix 2. There were a few small differences, which we mention in the results.

### Event-by-event predictive strength

**Fig. 2:**
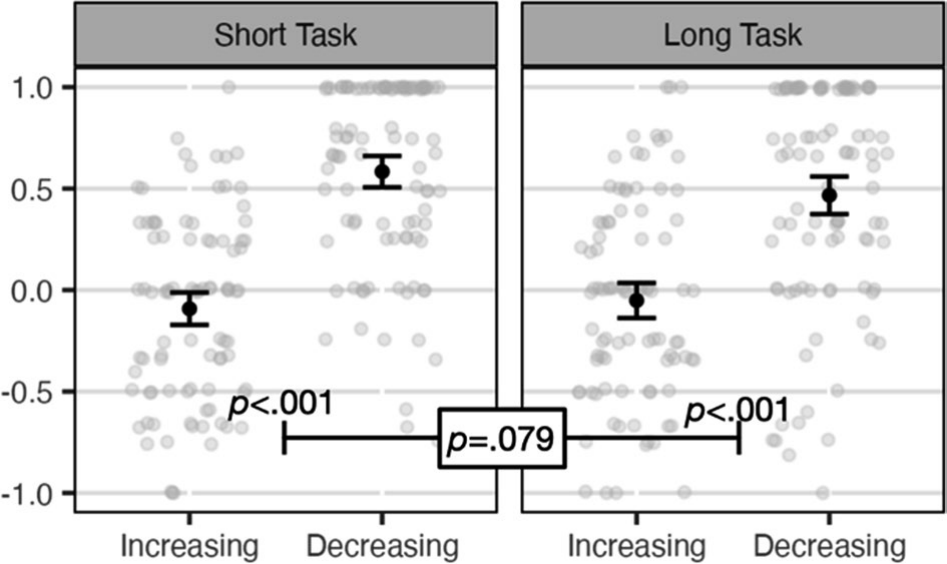
Event-by-event predictive strength. Note. Individual observations with jitter in gray. Means and 95% CIs in black. *P* values shown for increasing versus decreasing within the short and long tasks, and the interaction between increasing versus decreasing and task length

Before analyzing the main questions for this study, we first wanted to make sure that participants were able to learn the cause–effect relation in the first half of the study to confirm that the learning paradigm worked as expected; if not it would be difficult to interpret any of the subsequent findings. This analysis was not preregistered. The event-by-event predictions during Events 7–12 (Fig. 2) clearly show that participants in the increasing condition (who experienced a zero contingency during Events 1–12) gave lower predictions than those in the decreasing conditions (who experienced a positive contingency during Events 1–12). This was true for both the short task, *p* < 0.001, *BF* > 10^14^, *Cohen’s D* = 1.47, and the long task, *p* < 0.001, *BF* > 10^7^, *Cohen’s D* = 0.98. An ANOVA comparing the two timeframes revealed a marginal p-value of 0.079, *η*_p_^2^ = 0.02, suggesting somewhat better learning in the short timeframe, however, the BF of 0.49 was in the direction of the null. In sum, participants were able to learn the initial contingency in both conditions.

### Awareness of change

**Fig. 3:**
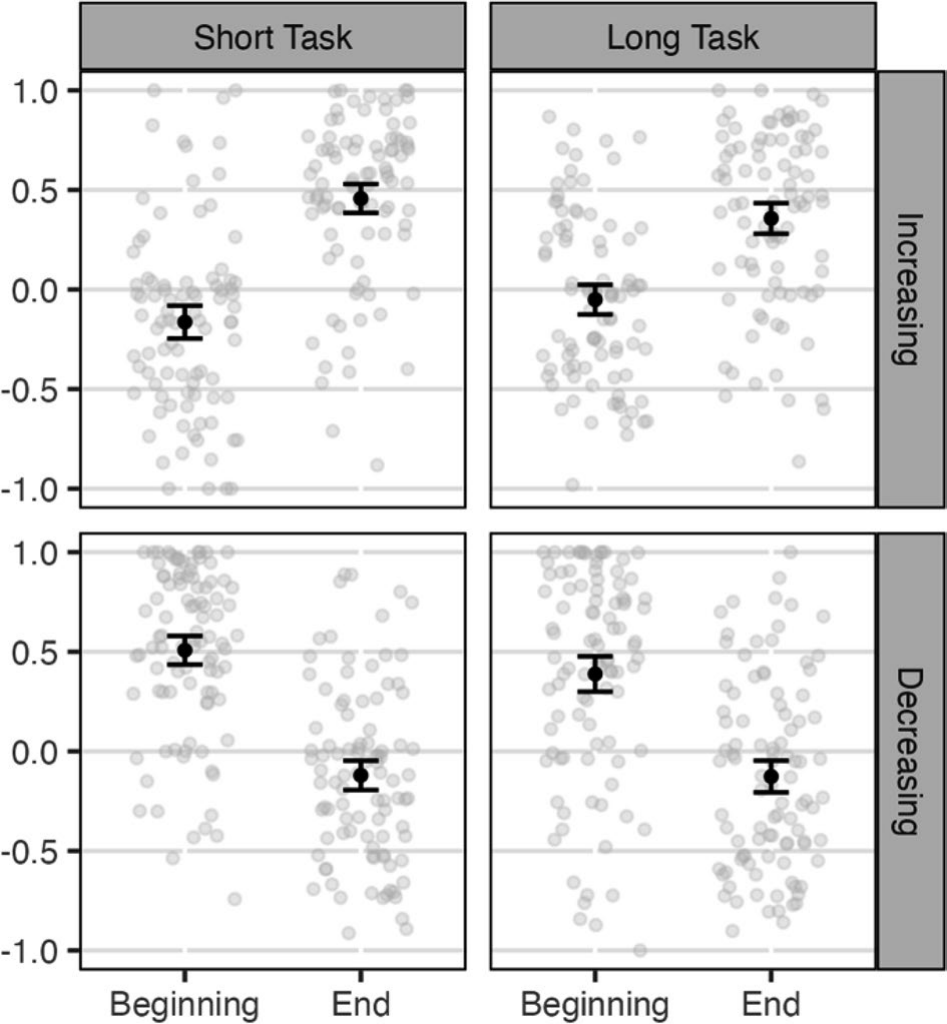
Awareness of change. Note. Individual observations with jitter in gray. Means and 95% CIs in black. There were significant 2 × 2 interactions, meaning that participants were aware of the change, within both the short and long task, *p*s < .001. There was a significant 3-way interaction, *p* < .001, meaning that the participants were more aware of the change in the short task

Figure 3 shows clear evidence that participants noticed the change in contingency in both timeframes. Notice how in the increasing conditions, participants causal judgments for the beginning of the task were near zero, and increased when asked about their more recent experiences. In contrast, in the decreasing conditions the opposite patterns occurred. To assess whether the increasing and decreasing conditions were different, evidence that participants learned the different contingencies, we ran A 2 (contingency change; between) × 2 (beginning vs. end; within) ANOVA. The interactions were reliable for both the short timeframe, *p* < 0.001, *BF* > 10^31^, *η*_p_^2^ = 0.48, and the long, *p* < 0.001, *BF* > 10^16^, *η*_p_^2^ = 0.29.

A 2 × 2 × 2 ANOVA was run adding on task length as a within-subject variable. The 3-way interaction was significant, implying that participants were more aware of the change in the short timeframe, *p* < 0.001, *η*_p_^2^ = 0.03, though the BF was only 2.36.

### Summary measures

**Fig. 4:**
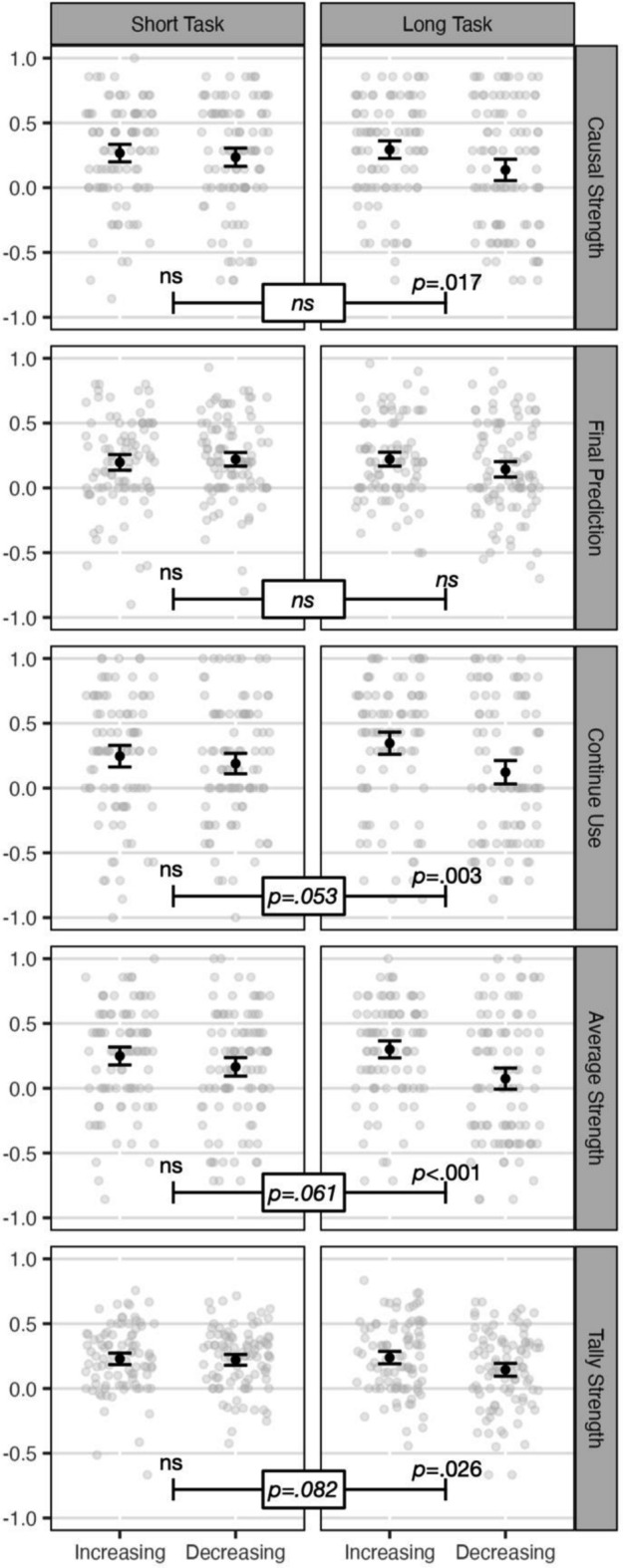
Summary measures. Note. Individual observations with jitter in gray. Means and 95% CIs in black. ns: *p* > .10. *p* values shown for increasing versus decreasing within the short task and long task, and the interaction between increasing versus decreasing and task length

This section reports the findings of the five summary measures, which speaks to whether there were primacy or recency order effects. Figure 4 shows plots and Table 2 provides the inferential statistics. Participants tended to provide positive judgments on average, which reflects the fact that the overall contingency was positive (the medicine worsened the side effect). A recency effect would comprise more positive judgments in the increasing condition, and a primacy effect would comprise more positive judgments in the decreasing condition.

**Table 2:** Inferential statistics for summary measures

Measure	Short timeframe			Long timeframe			Timeframe as moderator		
	*p*	*D*	BF	*p*	*D*	BF	*p*	*η*_p_^2^	BF
Causal strength	.596	− .08	.18	.017	− .35	2.27	.123	.01	.38
Final prediction	.623	.07	.18	.110	− .23	.52	.117	.01	.49
Continue use	.415	− .12	.22	.003	− .43	8.75	.053	.02	.56
Average strength	.166	− .20	.39	< .001	− .52	44.87	.061	.02	.55
Tally strength	.836	− .33	.16	.026	− .33	1.62	.082	.02	.48

For each measure, we conducted between-subjects *t*-tests to test for differences between the increasing and decreasing conditions in each timeframe separately. Additionally, we conducted a 2 (contingency change; between) × 2 (task length; within) ANOVA to test for whether task length moderated the effect of contingency change.

There were four key findings. First, there was no evidence of primacy effects. Second, in the short timeframe, none of the measures produced significant order effects. The BFs were all between 2:1 and 6:1 in favor of the null.

Third, in the long timeframe, four of the five measures produced significant recency effects, though there was a wide range of BFs. In the between-subject analyses (Online Appendix 2), Tally Strength was not significant, and Final Predictive Strength was.

Fourth, none of the five tests of differences between the short vs. long timeframe were significant. Though some were close to being significant, the BFs were still in the direction of the null. In the between-subject analyses (Online Appendix 2), three of these tests were significant, with BFs of 1.72, 3.43, and 8.73 for Final Predictive, Average, and Continue Use, respectively, suggesting that perhaps the difference between the short vs. long timeframe is reliable for some measures.

### Memory

#### Primacy and recency effects in contextually bound episodic memory

**Fig. 5:**
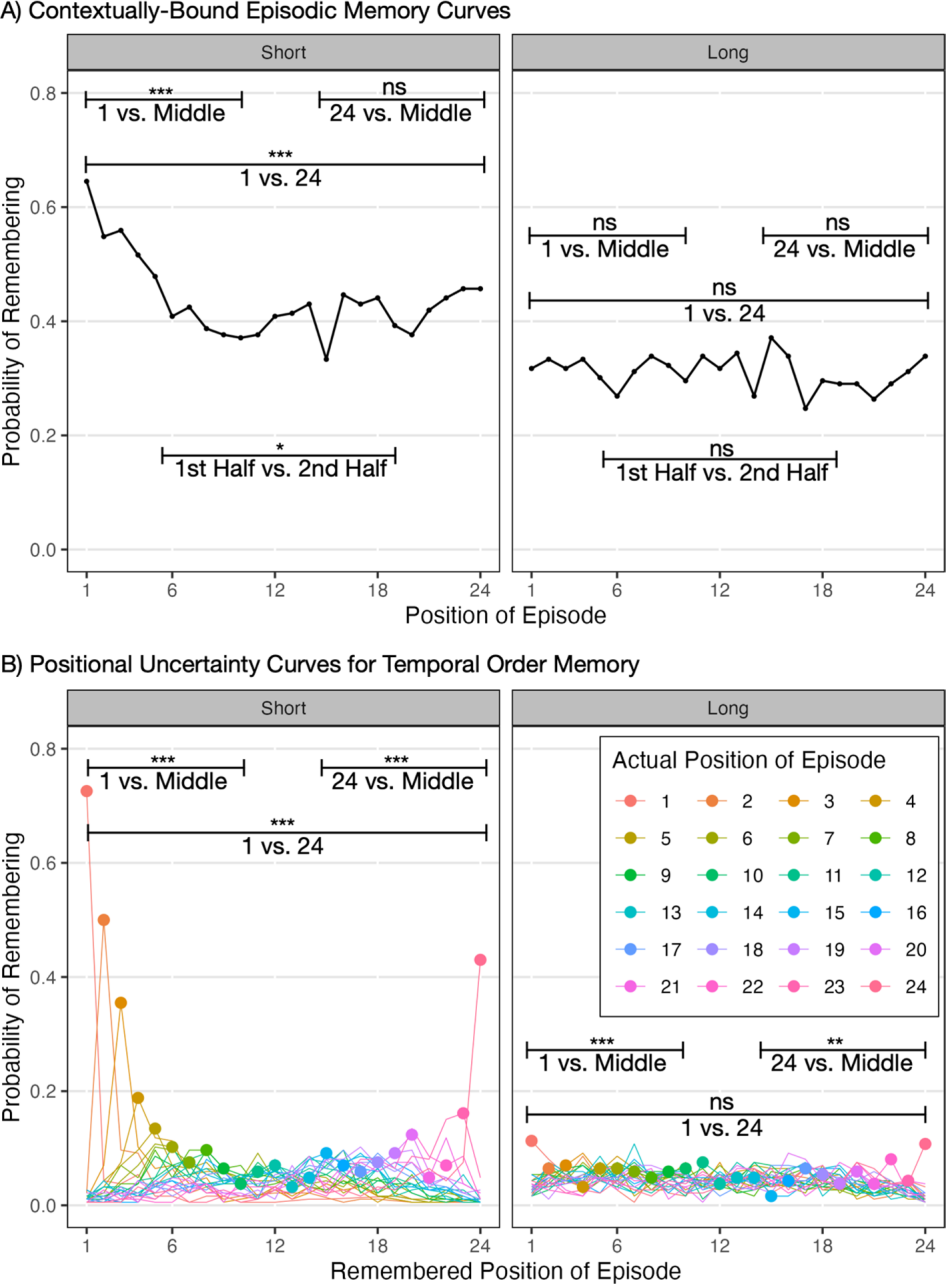
Memory analyses. Note. *p < .05. **p < .01. ***p < .001

Next, we tested for item order effects participants’ contextually bound episodic memories (Fig. 5a). In contrast to the summary judgments, it is possible to have both primacy and recency effects in memories for individual items. These analyses were conducted with random effects logistic regressions to test for the effects within the short and within the long timeframe, and an additional regression to test for differences between timeframes.^7^

The primacy effect was analyzed by comparing Episode 1 vs. the middle Episodes 6–19. There was a primacy effect in the short timeframe, *p* < 0.001, but not the long timeframe, *p* = 0.851, and timeframe was a moderator of primacy, *p* < 0.001.

The recency effect was analyzed by comparing Episode 24 vs. the middle Episodes 6–19. There was not a significant recency effect in the short timeframe, *p* = 0.122, nor long, *p* = 0.419, and timeframe did not moderate recency, *p* = 0.666.

The size of primacy vs. recency was analyzed by comparing Episode 1 versus 24. The primacy effect was larger than recency in the short timeframe, *p* < 0.001, but not the long, *p* = 0.638, and timeframe was a significant moderator, *p* = 0.003.^8^

Finally, all of the above analyses that tested timeframe as a moderator also included timeframe as a main effect. All found significant main effects such that participants’ memories were more accurate in the short timeframe than the long, *p*’s < 0.001.

#### Primacy and recency effects in temporal order memory

We also analyzed participants’ temporal order memories for primacy and recency effects. A standard way to plot data from order reconstruction tasks is with a graph of positional uncertainty curves (Fig. 5b). This figure shows for each of the 24 learning episodes, the likelihood that they were remembered to have occurred in each position. It is clear in the short timeframe that for the first few episodes and the last episode that participants had fairly good memories of when they occurred, and if they were wrong, they often guessed a nearby position. In contrast, participants’ memories in the middle were much more noisy and the errors were more spread out. In the long timeframe, all of the memories were fairly noisy.

We ran the same analyses as we did for the contextually bound episodic memories, and counted a memory correct if the participant chose the exactly correct position.^9^ There was a primacy effect in the short timeframe, *p* < 0.001, and the long timeframe, *p* < 0.001, and the primacy effect was larger for the short timeframe, *p* < 0.001. There was a recency effect for the short timeframe, *p* < 0.001, and long timeframe, *p* = 0.002, and the recency effect was stronger in the short timeframe, *p* < 0.001. Finally, the primacy effect was larger than recency in the short timeframe, *p* < 0.001, but not the long, *p* = 0.972, and timeframe was a significant moderator of this effect, *p* < 0.001.

#### Whether contingency change is reflected in contextually bound episodic memories

This final analysis of participants’ memories examines whether participants have contextually bound episodic memories that capture the change in contingency; this analysis was not preregistered. To assess this, we applied the ΔP formula, *p*(effect = present | cause = present) − *p*(effect = present | cause = absent), to transform participants’ memories of the 12 events in each half into a measure of the strength of the cause–effect relation in each half. This analysis is similar to the awareness of change measure, except that whereas the awareness of change measure assesses whether participants are generally aware of a change, this assesses if their episodic memories for events in the first half are different from their memories of events in the second half.

In the short timeframe, participants’ ΔP calculated from their episodic memories differed by 0.17 on average for the strong half vs. the no-contingency half. Though this is far from the ideal value of 0.67 if participants had perfect memory, it is significantly above zero, *p* < 0.001, BF = 3.9 × 10^3^, *D* = 0.36, implying that their episodic memories do capture the contingency change to some extent. In contrast, in the long timeframe, the difference was only 0.04 on average, which is not different from zero; *p* = 0.167, BF = 0.21, *D* = 0.10. Furthermore, the difference in the long timeframe was larger than in the short timeframe; *p* = 0.004, BF = 4.79, *D* = 0.22.

## Discussion

In the current study, we tested for order effects in participants’ causal judgments and memories, in both a traditional short timeframe task and in a more realistic timeframe. In the introduction, we posed three key questions. In the following sections, we discuss findings related to each of these key questions. Table 3 summarizes the findings, and the results in Table 3 can be compared against the predictions made by the three theories in Table 1. Afterward, we discuss other implications for theories of causal learning and judgment and theories of episodic memory.

**Table 3:** Summary of findings for the three questions

Questions	Short task	Long task	Difference between short versus long
Q1. Aware of change	Yes, aware of the change	Yes, aware of the change	Participants were significantly less aware in the long timeframe
Q2. Summary measures of cause–effect relation	Roughly average over all learning episodes. (Neither primacy nor recency.)	Mainly recency	Some of the differences between conditions were marginally significant
Q3. Episodic memory	Strong primacy and recency effects. Primacy stronger	Small primacy and recency effects, and only for one measure	Primacy and recency effects were significantly weaker in the long timeframe

### Question 1: participants were aware of the change in causal strength

Question 1 asked whether participants would be aware of the change in strength in both the short and long timeframe tasks, and whether there would be a difference in timeframes. Indeed, we found that participants could detect and recall the difference in contingencies at the beginning and end of the learning sequence, in both short timeframe and long timeframe situations, though somewhat better in the short timeframe. This finding is important because in real-world environments contingencies can change, and detecting this change is useful for making decisions for the future.

From a theoretical perspective, an important question is how are people able to notice a change in contingency. As explained in the introduction, of the three standard models of causal learning, only episodic memory could support noticing a change in strength. However, our findings do not fit well the possibility that episodic memory is primarily supporting noticing a change. First, participants’ episodic memories were not all that accurate except for the first few episodes and the last episode. Second, when we analyzed if contingency change is reflected in contextually bound episodic memories, the change was fairly subtle though significant in the short timeframe, and was not significant in the long timeframe. In contrast, when participants were simply asked about the relationship, they were able to much more accurately say that the strength of the relationship at the beginning was different from the strength at the end, even in the long timeframe. The episodic memories may be providing some insight into contingency change, particularly for the short timeframe, but clearly participants’ awareness of the change is not dependent on their episodic memories.

From a more practical perspective, the fact that participants noticed the change has implications for how to interpret the summary measures. In prior studies on order effects in causal learning, participants were not asked if there was a change in contingency, just summary measures. Since we found that participants were very clearly aware of the change in both the short and the long timeframes, this raises the possibility that item-order effects in summary measures may be impacted by demand characteristics of how participants think they are supposed to answer the questions; if people learn about a relationship that changes dramatically across the dataset and are asked to make a summary judgment about it without being given an option to note this salient change, they may grapple with how to answer the question. In sum, having summary measures alone without a measure of awareness of change makes it hard to know how to interpret the results.

### Question 2: order effects in causal learning

The second key question for this research is whether there are order effects in summary judgments about the cause–effect relation, and whether there are differences between the short and long timeframe. Prior research in short timeframes has sometimes found primacy, and sometimes recency, and more broadly these effects tend to be fairly modest. In the introduction we provided reasons why the long timeframe could lead to more of a recency effect, or more of a primacy effect, or no difference compared to the short timeframe.

None of the summary measures produced a significant order effect in the short timeframe. In fact, most of them had BFs roughly in the range of 5:1 in favor of the null hypothesis. It is not accurate to say that our lack of finding an order effect is inconsistent with the prior literature since the prior research has been variable and the effect sizes have typically been modest.

In contrast, we found a number of significant recency effects in the long timeframe, with a wide range of BFs. It is uncertain whether there is a reliable difference between the short vs. long timeframe; none of the comparisons in the main analysis were significant (*⍺* = 0.05) though three were significant in the between-subject analysis (see Online Appendix 2). It is possible that with larger samples, the pattern of recency effects and differences between the two conditions would be more reliable, though the shift appears to be relatively small.

In the introduction, we posed the hypothesis that if participants are aware of the change in contingency, then questions that ask about making predictions or decisions about the future (Final Predictive Strength and Continue Use) may exhibit a recency effect whereas other measures may not. The reason is that focusing on recency might be adaptive from the perspective of making decisions in a changing world. However, it does not appear that participants selectively focused on recent experiences only for these measures; the recency effect was reliable for Continue Use in the long timeframe, but not Final Predictive Strength, and there was a reliable recency effect for Average Strength in the long timeframe.

Whether or not a participant noticed the changing contingency could also, in theory, impact how they answer Causal Strength vs. Average Strength. If one notices the change in strength, they might interpret Causal Strength to be asking about the more recent events, as this could be useful for making decisions for the future. In contrast, Average Strength explicitly said to weigh all the experiences equally. In the short timeframe participants did not exhibit order effects for either question, and in the long timeframe they exhibited a recency effect for both. Thus, contrary to this hypothesis, in the current study it appears that people interpreted them fairly similarly. (In fact, the recency effect in the long timeframe was numerically stronger for Average Strength than for Causal Strength, the opposite of this hypothesis.) In sum, it is still unclear how people interpret the commonly used Causal Strength question, and what factors may lead to more of a primacy or recency effect.

Overall, one read of the pattern of results is that there may be a general shift toward recency in the long timeframe. However, there do not appear to be clear reasons for why some measures exhibited this shift more than others, and any shift toward recency in the long timeframe is fairly modest. One lesson from this research is that asking multiple dependent measures, especially ones intended to get at participants’ awareness of whether or not the contingency changes, is important for having a fuller understanding of order effects in causal judgments.

### Question 3: similarities and differences between causal judgments and item memory

Question 3 asked whether order effects in summary causal judgments and episodic memory were similar or different, which can shed light on whether episodic memory may underly causal judgments. Table 1 summarizes the predictions of the three cognitive processes hypothesized to underlie causal inference and Table 3 summarizes the results.

In the short timeframe there were significant primacy effects for both memory measures, recency for one, and primacy was stronger than recency for both. In the long timeframe, the memory curves were much more shallow. Furthermore, these primacy and recency effects were significantly stronger in the short timeframe than long for five out of six tests.

Overall, when comparing the findings for the summary measures in Question 2 vs. the episodic memories in Question 3, there are striking differences. In the short timeframe, there were no order effects for the summary measures, whereas for the episodic memories primacy was considerably larger than recency. In the long timeframe, there were recency effects in some of the summary measures, whereas there were not reliable differences between primacy vs. recency in the long timeframe episodic memories. Summarized a different way, the summary measures appeared to show a trend toward recency with the longer timeframe compared to short, whereas the episodic memories appeared to show worse memory and smaller item order effects in the long timeframe compared to short. Thus, our current findings do not provide much evidence that people’s causal judgments are closely tied to contextually bound episodic memories.

We are not arguing that episodic memory plays no role in causal judgment. It is clear that participants did have contextually bound episodic memories for some events, particularly at the beginning. And it is still possible that certain key episodic memories influence the causal judgments. But the different patterns across the memories and causal judgments suggest that episodic memory is not the primary process in causal learning and judgment.

One question for future research is that in some real-world situations people may have especially strong, rich, and contextually bound episodic memories; the episodes in the current study were short and not as personally-meaningful as many naturalistic events are. For example, when starting a new medication, one might have some strong episodic memories of taking the medicine and outcomes, and likewise for other meaningful changes in one’s life.

Another possibility is that even if our participants did not have strong contextually bound episodic memories, that they may have still had non-contextual episodic memories of the cause and effect and used these for making causal judgments. If so, these would be hard to measure, and a theory in which people have non-contextually bound episodic memories (memories of the cause and effect that are not tied to a contextual image nor tied to the time when it occurred) would be hard to distinguish from storing tallies. An important goal for future research is to better understand the connections between episodic memories, memories for tallies, reinforcement learning, and causal judgments.

### Implications for theories of causal learning and judgment

Earlier in the discussion we already commented on one main implication for theories of causal learning and judgment—that there is little evidence that people rely on episodic memory for making causal judgments. In this section, we discuss implications for reinforcement-learning and tally-based theories of causal learning and judgment.

Standard reinforcement and associative learning accounts (e.g., Rescorla & Wagner, 1972) assume that people simply update their belief about the strength of the cause–effect relationship after each event. Such models are compatible with our finding of some recency effects in the long timeframe; most RL models produce recency effects (though this is not necessarily the case in all situations if learning rates change over time, e.g., Danks, 2003). However, RL models do not inherently predict more recency in long timeframes. They could potentially accommodate this if there was a faster learning rate parameter in the long timeframe than short; however, in prior research we found few differences in acquisition between short vs. long timeframes, and if anything, a hint at faster learning the short timeframe (Willett & Rottman, 2021). Additionally, as mentioned above, standard model-free RL accounts are incompatible with the finding that participants are aware of the change in contingency, though more sophisticated RL models that infer latent states (e.g., Courville et al., 2006; Daw et al., 2008) could in theory do this. We believe that these more sophisticated theories offer a promising approach to causal learning that should be further investigated.

“Rule-based” models of causal learning (e.g., Cheng, 1997; Griffiths & Tenenbaum, 2005; Hattori & Oaksford, 2007) assume that people remember tallies of all the event types. These models do not predict item order effects because they do not make assumptions about memory. One particular challenge for these models is that in their current forms, they do not have a mechanism for detecting a change in contingency or for keeping separate tallies when a change is noted. In contrast, the current results clearly show that people notice such changes and can fairly accurately recall the contingency during each period. Thus, such models would need to be modified to account for understanding changing causal strength.

It might be tempting to think that it would be easy to modify tally theories to be able to notice a change; however, we suspect that such a modification is not viable. From our current findings, we are assuming that people spontaneously track more distant and more recent causal strengths, and that at any point in time they have access to both. To be precise, consider an example of how this would work if people continuously monitor the first half and second half of experiences. This example is not being proposed as what could literally occur in the mind; the purpose of the example is to demonstrate how hard this would be. Suppose that the learner has already experienced 11 episodes, so has a tally for the initial 5 and the latter 6 in memory. Then they experience a 12th episode; upon experiencing the 12th, they would have to remember what type of experience the 6th episode was, and remove it from the latter set of tallies and add it to the initial set of tallies. This means that at any given time the learner would need to have fairly accurate memories for the entire second half of experiences; they cannot just keep a tally for the second half because in order to shuffle the middle episode (the 6th in the prior example) from one tally to the other they need to remember this episode. Of course this proposal is not realistic in a variety of ways, but it strikes us that there is not an easy solution to this problem from a tally-based perspective. And to the extent that more distant experiences are more noisy in memory, this updating process would be less accurate at detecting changes between the first and second half. One way to make this process much easier would be if the learner keeps episodic memories of the last few episodes, and then a tally of all the prior episodes. However, this process would only allow one to detect very recent changes in causal strength, not changes that occurred longer ago. In sum, given that our participants were quite accurate at detecting changes between the first 12 and last 12 learning episodes, but that their memories for specific events was quite poor, this sort of process does not seem to be a viable explanation to us.

One intriguing future direction is to consider Hintzman’s recursive-reminding theory (2004, 2010) as a bridge between episodic memory and tallies. Hintzman proposes that when people experience a repeated events (e.g., cause-present effect-present), they are reminded of the prior experience; these experiences build on each other recursively, which can form a basis for judging the frequency of occurrences as well as the spacing of the repetitions. Potentially this sort of representation could help a learner notice that certain types of causal events were more present earlier on and less present later, and vice versa. That said, this theory is based on episodic memories and remindings of memories, and as already discussed, our participants’ episodic memories were not all that strong, at least not the way we assessed them as tied to contextual images. The recursive-reminding theory is currently only qualitative; it could be productive to implement a process model to help to clarify whether it is a viable explanation for our findings.

Stepping back to consider which of these three main theories, tallies, reinforcement learning, and episodic memory, fit best with the current results, we do not believe that any of these theories is a particularly good fit. Episodic memory predicts different order effects than found in the summary judgments, and tallies and reinforcement learning do not predict that participants are aware of a change in contingency. One interpretation of the current results is that the shift toward recency for the summary judgments in the long timeframe may be a sign of more reliance on RL processes in the long timeframe. However, given that participants were still able to notice the change in contingency in the long timeframe, this is not a complete explanation. It is clear that there are still fundamental open questions about how people learn causal relations from experience and make judgments. Though it is possible that there is another parsimonious theory that explains all of these findings, it is also possible that people in engage in all three of these processes during learning and then rely on different ones or combinations of them for making inferences.

### Implications for theories of memory

This research has primarily been framed as about causal learning and inference, and has used episodic memory as a potential explanation for causal inference. However, this research also presents a unique test of episodic memory in its own right. Item order effects in memory are typically studied with unique items that do not repeat (e.g., lists of words). In contrast, in our study, though the contextual images were unique, the presence/absence of the cause and effect were repeated, which is related to two strands of research in the memory literature. First, there is a line of research on ‘personal semantics’ (e.g., where did I park my car today, did I remember to make my medicine today, did I water the plants this week; Renoult et al., 2012, 2016), which is somewhat similar to the repeated events in causal learning (e.g., on days that I took the medicine did I experience different outcomes compared to days that I did not take the medicine). Second, there is a line of research on memory for ‘repeated’ events, for which (e.g., Rubínová & Price, 2024) some features of the events are repeated and some may be unique.

Research on item order effects in memory is vast and touches on some of the core debates in memory such as whether there are separate short-term and long-term memory stores or a single storage system (Brown et al., 2007; Davelaar et al., 2005, 2006; Sederberg et al., 2008; Usher et al., 2008). It is beyond the scope of this paper to discuss all the relevant findings and theories, but we will bring up a few issues that are especially relevant.

In item order effects research, there are a number of factors that have been shown to moderate the size of the primacy effect and the size of the recency effect. Dual-store theories tend to propose that recency effects are due to simple read-out of the short-term memory buffer, so if the test is postponed (retention interval is lengthened) or if there is a distraction between the learning and test and the recent items are no longer stored in short-term memory, the recency effect is reduced (Atkinson & Shiffrin, 1968, 1971; Davelaar et al., 2005). In contrast, single-store theories propose that instead of just the retention interval mattering, it is really the ratio between the interpresentation interval (time between the learning items) and the retention interval that matters (Bjork & Whitten, 1974; Glenberg et al., 1983). If the retention interval is lengthened holding the interpresentation interval constant, this can decrease the recency effect and increase the primacy effect (e.g., Neath, 1993; Wright et al., 1985). Additionally, the ratio rule predicts that if the entire sequence of learning and retrieval events are spaced out proportionally, that there should not be any differences in the recency effect in free recall. More specifically most single-store models rely on the relative distinctiveness of memory traces of the learning items, which is impacted by both this ratio and edge effects (Brown et al., 2007).

It is useful to consider our results in comparison to Mack et al.’s (2017) smartphone studies, which used one hour interstimulus intervals for word lists, with free recall testing at the end of each day, for many days. Most importantly, Mack et al. argued that both the primacy and recency effects were smaller than what are typically found in short timeframe studies, and is not consistent with the ratio rule.

Though there are a number of differences between our studies, we found a similar pattern of diminished primacy and recency effects in the long timeframe compared to the short. In Online Appendix 1, we discuss our findings in relation to the ratio rule; we provide details of the interpresentation interval and retention interval that individual participants experienced, and we modeled the primacy and recency effects with Brown et al. (2007) model. Like Mack et al. we also conclude that our findings are not consistent with the ratio rule. At the same time, we acknowledge that the nature of the stimuli we used, particularly the repeated nature, is not common in research on memory, nor is the paradigm we used of only one learning item per day for many days, so it is somewhat challenging to compare our findings to existing research. More broadly, we believe that our paradigm of presenting items via smartphone spaced out over many days may be useful to test theories of memory that are hard to test otherwise.

### Methodological considerations

#### The learning data

Our study used a less dramatic change in contingency compared to prior studies. In many prior studies, the cause–effect relation changed from strongly positive to strongly negative, or vice versa, whereas in the current study it changed from positive to zero, or zero to positive. We used a less dramatic change in contingency compared to prior studies for three reasons.

First, in most prior studies the base-rate of the effect occurring when the cause was absent changed from one half to the other, which results in a dramatic shift in the distribution in the four types of events. As explained in the methods, we decided to have the strength of the cause–effect relation change but to keep the base-rate of the effect when the cause was absent the same across the two halves. The reason for this choice is that it seems too coincidental and unexplainable that both the strength of the cause–effect relation and the base rate of the effect would change at the same time. It may be fruitful to investigate whether these two approaches to investigating order effects (only one changing vs. both changing) produce different order effects.

Second, in many prior studies the change from one half to the next was so strong that it was somewhat inconceivable to us that participants would not notice it, at least in the short timeframe. We thought that testing whether they notice the difference in a somewhat subtle case would be a more realistic assessment of real-world causal learning.

Third, though we do not have evidence to support this view, it seems to us to be more common for a cause to get stronger or weaker, but not cross from being positive to negative. Though we acknowledge that causes can change polarity, this seems less common and more artificial, and one of the main goals of this study was to increase the external validity of the design.

#### Judgments during learning

In this study participants predicted whether the effect would be present or not during each learning episode. Prior research has found that when participants make repeated judgments during learning they tend to exhibit a recency effect, whereas if they just make one final judgment they tend to exhibit averaging or primacy (Catena et al., 1998; Collins & Shanks, 2002; Glautier, 2008; Marsh & Ahn, 2006; Vadillo et al., 2004; see also Hogarth & Einhorn, 1992). These papers raise a number of potential explanations for this finding: when the question is asked repeatedly participants might interpret the question as asking specifically about the more recent experiences, it could prevent a decrease in the learning rate that could otherwise occur due to decreased attention over time, and it could increase working memory demand and lead toward more use of RL processes.

Since participants made predictions on each trial, it is possible that this could explain some of the recency effects that we found. At the same time, participants made predictions in both the short and long timeframes, so this would not explain differences between timeframes. We doubt that it has a strong impact on the memory findings or the ability to notice the change in causal strength, though in theory it could.

## Conclusions

This study investigated how people learn about a cause–effect that changes in strength over time. First, we found that participants were very capable of noticing a change in the contingency between the cause and effect, though the process used to do so remains unclear. Furthermore, this ability and calls into question prior studies on order effects in causal learning and other fields that ask participants to make a summary inference (e.g., judging an individual’s personality or a defendant’s guilt) without giving them the possibility of reporting that they noticed a change; conclusions from such studies may need to be re-examined and re-interpreted.

Second, for the causal judgments, we found some evidence of recency effects in the more ecologically long timeframe condition, but no primacy or recency effects in the short timeframe.

Third, we found evidence of both primacy and recency effects in participants’ episodic memories of individual cause–effect events in the short timeframe. In the long timeframe, the primacy effects were weaker but still present, and the recency effects were no longer present. These findings suggest that the summary causal judgments are not primarily based on participants’ episodic memories.

Lastly, building on Mack et al. (2017), this research extends order effect research into more real-world timeframes. We hope that this study inspires other researchers to use ecological momentary experiments as a method for studying causal learning and memory in more realistic settings.

## Supplementary Information

Supplementary Material 1.

Supplementary Material 2.

## Data Availability

The study’s analysis plan was registered after data collection. See https://osf.io/udwkx/ for the analysis plan, data, and analysis code. Study materials are available upon request.

## Footnotes

1 Here we are focusing on the standard versions of these theories. Alternative versions of RL models can be made that do not necessarily produce a recency effect, such if the learning rate decreases over time (Danks, 2003). Modifications to the Tally theories could be made, perhaps to incorporate retroactive or proactive interference, which could lead to order effects.

2 Some contingency learning studies have found what is called a “recency to primacy shift;” testing immediately after learning tends to exhibit a recency effect, whereas after a delay responses exhibit a primacy effect (Stout et al., 2005; see also Wheeler et al., 2004, for a similar study using a conditioning paradigm with rats, and see Pineño, & Miller, 2005 for a review of primacy and recency in conditioning research). However, one considerable difference between these studies and ours is that those studies simply lengthened the retention interval (time between learning and test), whereas in our study both the interstimulus intervals and retention interval were increased from the short to the long timeframe. Thus, this phenomenon does not directly apply to the current research.

3 We chose 24 because is the number used by Willett & Rottman (2021). Also, if there were considerably fewer days, it could start to be challenging to get the contingencies in the range we wanted, and if there were many more it would increase the difficulty of getting participants to complete the study.

4 The long timeframe task started on Day 5 rather than Day 2 so that the entire study would end exactly 4 weeks after it began, making it easier for participants to schedule visits to the lab.

5 For one participant in the short condition and one participant in the long condition calculating ΔP from Events 7–12 resulted in a divide by zero error. This occurred because, merely due to the randomized order, the cause happened to be present or absent for all six events. For these two participants, we expanded the range to include one more event (Episodes 6–12), the minimal change possible, to calculate ΔP.

6 We asked the questions in this order for the following reasons. We asked the causal strength question first because it is the most standard dependent measure in studies of causal inference, and we wanted an untarnished measure. We asked final predictive strength and continue use prior to awareness of change because we felt that if awareness of change was asked first it could affect how participants answered these two questions, perhaps leading to stronger recency effects or lead participants to second guess these straightforward questions. We asked about tallies last because it is the most objective and least likely to be affected by other questions.

7 We started with random slopes for all three predictors. If the model did not converge, we followed Barr et al.’s (2013) suggestions to simplify the model. We removed the correlations between the random slopes. If this did not converge, we removed the random slopes on the interaction. If that did not work, we removed the random slope on the timeframe. The code available on OSF details which models converged for each analysis. The interactions with timeframe were registered. We followed up these analyses with separate regressions within each timeframe, which were not included in the registration. BFs and effect sizes were not calculated since these regressions used random effects and these are hard to calculate for models with random effects in standard packages. However, since the dependent measure is binary comparing the means provides an interpretable understanding effect size.

8 Another way to compare primacy vs. recency is to compare the memories for the entire first half vs. the entire second half; comparing memories for the two halves is useful because it appears that the improved memories were not just present for the very first and very last episodes, but potentially for a few more near the beginning and end. Furthermore this analysis is also revealing because if episodic memories are used for making summary judgments, then the memories for the entire two halves should reflect the same order effects as the summary judgments. To test this, we conducted similar repeated measures logistic regressions to the prior analyses, but now comparing the two halves (Episodes 1-12 vs. 13-24); this analysis was not preregistered. Participants had significantly better memories for the first half than the second half within the short timeframe, *p* = .016. However, there was no difference between the halves in the long timeframe, p = .377, and the interaction with timeframe was not significant, *p* = .273. There was a main effect of timeframe, *p* < .001.

9 We also considered other metrics to allow for nearly-correct responses to be counted as correct as well. These alternatives did not change the general findings, and introduce some analytical challenges. Thus, we decided to use this analysis.
