# Supplementary material for: Learning about causal relations that change over time: primacy and recency over long timeframes in causal judgments and memory
Source: Cogn Res Princ Implic. 2025 Feb 21;10:9. doi: 10.1186/s41235-025-00614-9 (PMC11845336; doi:10.1186/s41235-025-00614-9)
Supplement: Supplementary file 1 — Supplementary Material 1. [file 41235_2025_614_MOESM1_ESM.pdf]

Online Appendix 1: Additional Details Pertinent to Memory for Individual Items

Timing Data of Memories, The Ratio Rule, and Timeframe Invariance

The ratio rule states that the size of the recency effect in free recall studies is proportional to the ratio of the interpresentation interval (IPI; time between each learning item) to the retention interval (RI; time between learning and recall) (e.g., Bjork & Witten, 1974; Crowder, 1976, 1993; Nairne, Neath, Serra, & Byun, 1997). The ratio rule predicts that if the entire sequence of learning and retrieval events are spaced out proportionally, that there should not be any differences in the recency effect in free recall. Though the ratio rule has often been specifically proposed for free recall, which we did not measure, Brown, Neath, and Chater (2007, p. 540) argue that bowed serial position curves and timescale invariance are found for a variety of different memory tasks that we did measure (such as recognition memory and temporal location memory). Additionally, one of the main theoretical accounts of the ratio rule based on temporal distinctiveness (Brown, Neath and Chater, 2007) proposes timescale invariance – that if all of the timing of the events is proportionally spread out, then there should be no impact on memory. Thus, in this appendix we provide more details about the timing of the events in the short and long timeframes.

In our study the retention interval was not stretched out proportionally to the interpretation interval for the memory judgments. Doing so would have required each subsequent memory question to occur on a separate day rather than grouped together on the last day of the study. In the short task the IPI/RA is in the range of roughly 0.1, whereas in the long task the IPI/RA is close to 1.0; thus, the ratio rule predicts a larger recency effect in the long timeframe than the short, but in fact we found the opposite.

Online Appendix 3 Figure 1 shows histograms of some of the IPI, RI, and IPI/RI for both tasks. This analysis includes 147 of the participants; the data for 39 participants are not shown because some of their timestamps were not correctly recorded.

The interpresentation interval (IPI) is the average duration between events in the learning task. The IPI was peaked at about 15 seconds in the short timeframe. (The timing data were recorded only to the accuracy of a minute, so this was calculated by taking the time of the entire learning task and dividing by 24, hence the spaces between the bars.) In the long timeframe, the IPI was peaked at close to 24 hours. A few participants had IPIs near 25 or 26 hours; these are the participants who missed one or two days so their entire learning task took 25 or 26 days.

The retention interval (RI) is the time from the last learning episode to when that last item appeared in the testing phase. Between the learning and testing phase participants answered the summary causal learning questions, and since all 24 images were tested in a random order, depending on when the 24th item was tested the RI could be shorter or longer. In the short timeframe it was peaked at around 3-4 minutes. In the long timeframe, the RI was roughly 24 hours for most people, though a few people were tested on the same day as the last learning episode.

Online Appendix 3 Figure 1c shows the IPI/RI ratio, which prior research has shown predicts the magnitude of the recency effect. The ratio for the long timeframe is almost entirely peaked near 1 since the testing phase was roughly 1 day after testing for almost all participants. (There were 13 observations larger than 3, not plotted in the graph; these were participants who did the testing task on the same day as the last day of learning.) In the short timeframe condition the ratio was considerably smaller for almost all participants, and peaked at less than .1. The reason is that due to the intervening measures (causal judgments and other memory probes), the

RI is *relatively* much larger in the short timeframe; in the long timeframe the intervening measures add on a trivial percent of time on top of the 24 hour delay.

In sum, the IPI/RI ratio predicts a larger recency effect for the episodic memories in the long timeframe than the short. However, if anything we found larger or equivalent recency effects in the short timeframe than the long.

Online Appendix 3 Figure 1. Histograms of timing data for memory measures.

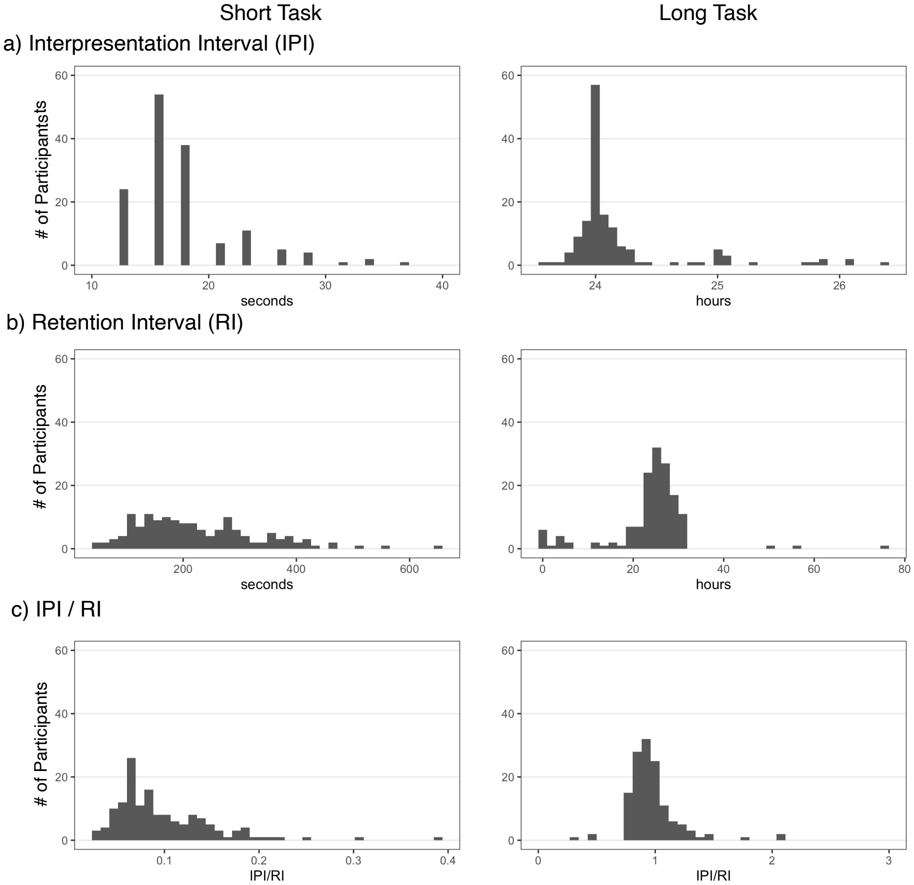

Simulation of SIMPLE

We also ran simulations of Brown, Neath, and Chater's (2007) SIMPLE model of memory retrieval, which was designed to capture primacy and recency effects in free recall and serial recall. Like the ratio rule, SIMPLE is timescale invariant, but can also account for primacy effects in addition to recency. We ran the serial recall version of the model to simulate participants' temporal order memories.

We ran simulations versions using different combinations of the three parameters, with a wide set of ranges and verified that the qualitative patterns do not change outside of these ranges. For the *c* parameter we ran simulations using 5, 15, 100, and 500. For the *s* parameter we ran simulations for 2, 5, 8, and 10. For the *t* parameter we ran simulations using .1, .5, .8, and 1. For the long timeframe, the simulated data assume that the ISI was 24 hours and that the RI was 24 hours; for the short timeframe, ISI was 15 seconds and RI was 200 seconds. (Simulations using

actual experienced timing data including times for the learning events and unique times for each retrieval in the test phase reveal very similar patterns.)

The simulations of SIMPLE reveal that, assuming that the three parameters are held constant for the short and long timeframes, then memory was always predicted to be better or equivalent for the long than the short timeframe condition. This deviates from our finding that memory was better in the short timeframe. That said Brown, Neath, and Chater (2007, p 565) discuss how one of the parameters, *c*, may depend on the timescale, which would complicate this analysis.

In summary, our simulations of SIMPLE do not fit our empirical findings well, most obviously with regards to the fact that our participants' memories were uniformly better in the short than long timeframe.

Online Appendix 3 Figure 2. Simulations of SIMPLE.

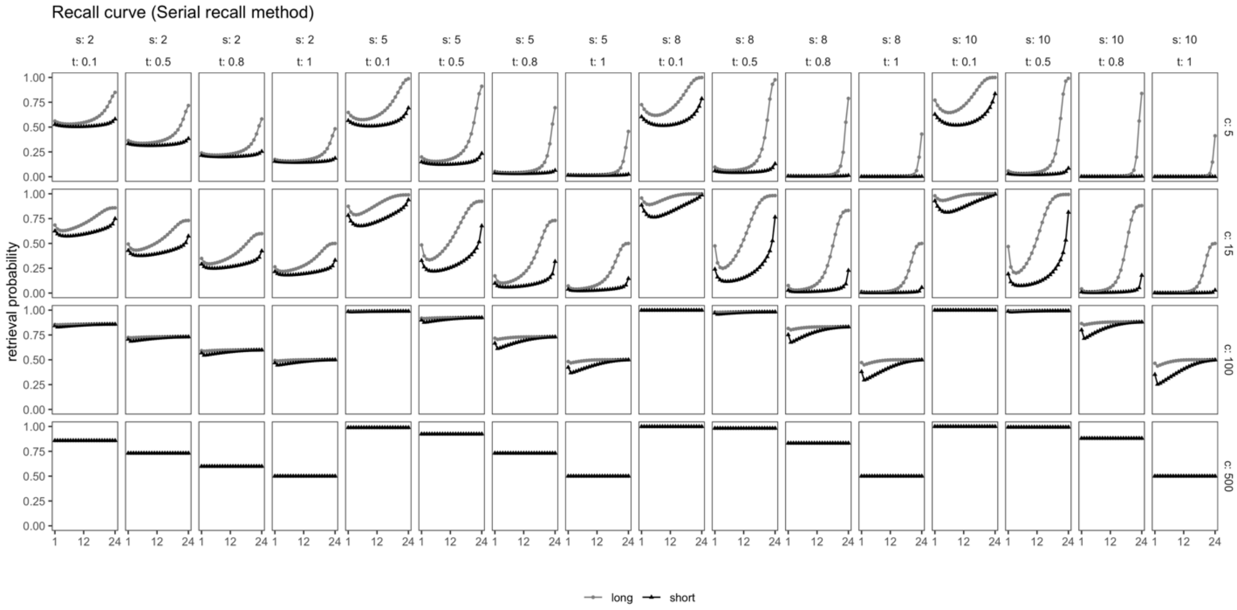

References

Barr, D. J., Levy, R., Scheepers, C., & Tily, H. J. (2013). Random effects structure for confirmatory hypothesis testing: Keep it maximal. *Journal of Memory and Language*, *68*(3), 255-278.

Bjork, R. A., & Whitten, W. B. (1974). Recency-sensitive retrieval processes in long-term free recall. *Cognitive Psychology*, *6*(2), 173-189.

Crowder, R. G. (1976). *Principles of learning and memory.* Hillsdale, NJ: Earlbaum.

Crowder, R. G. (1993). Short-term memory: Where do we stand? *Memory & Cognition*, *21*(2), 142-145.

Nairne, J. S., Neath, I., Serra, M., & Byun, E. (1997). Positional distinctiveness and the ratio rule in free recall. *Journal of Memory and Language*, *37*(2), 155-166.
