# Supplementary material for: Learning about causal relations that change over time: primacy and recency over long timeframes in causal judgments and memory
Source: Cogn Res Princ Implic. 2025 Feb 21;10:9. doi: 10.1186/s41235-025-00614-9 (PMC11845336; doi:10.1186/s41235-025-00614-9)
Supplement: Supplementary file 2 — Supplementary Material 2. [file 41235_2025_614_MOESM2_ESM.pdf]

Online Appendix 2: Additional Details for Summary Judgment Analyses

In accordance with our registration on OSF, we tested for an effect of task order - whether the fact that each participant did two tasks (both short and long timeframe) made a difference. To test this, we ran a three-way interaction between contingency change (between subjects: increasing vs. decreasing), timeframe (within subjects: short vs. long), and the order of the task (between subjects: completed short task first vs. completed long task first) for each of the dependent variables except for the memory measures. The results are in Online Appendix 2 Table 1. There was no significant three-way interactions or two-way interactions for any of the dependent measures, and most of the Bayes Factors provided at least moderate evidence in support of the null hypothesis (no effect of task order).

Out of an abundance of caution, we also ran the analyses from the main text using only the first task that participants completed, so it was a completely between-subjects design. Online Appendix 2 Table 2 reports the results of t-tests comparing the increasing vs. decreasing conditions. The results in the “All” columns correspond to the results shown in the main paper. The results of the “First” columns only use the data from the condition that each participant completed first. The All and First results are very similar; there are small differences in the results for Final Predictive and Tallies in the Long Timeframe.

Online Appendix 2 Table 3 reports the results of the 2 (contingency change) × 2 (task length) ANOVA to test whether the effect of contingency change was moderated by timeframe. The results in the “All” columns correspond to the results shown in the main paper. The results of the “First” columns only use the data from the condition that each participant completed first, so contingency change was between subjects. Though we report the main effects, the interactions are what we care about as they test whether there is a significant shift between primacy vs. recency across timeframes. All of the interactions are mathematically in the direction of more recency in the long task compared to the short task. Though none of the interactions are significant in the All analysis, three are significant in the First analysis, suggesting that perhaps the difference between the short vs. long timeframe is reliable.

Online Appendix 2 Table 1. ANOVAs Testing for an Effect of Task Order.

**Measure**	***F***	***p***	**η_p^2**	***BF***
**Contingency Change × Task Order Interaction**				
EBE Predictive	1.02	.314	.01	0.32
Causal	1.08	.300	.01	0.27
Final Predictive	1.80	.181	<.01	0.42
Continue Use	3.86	.051	.02	0.89
Average	2.28	.133	.01	0.63
Tally Strength	0.46	.497	<.01	0.22
AOC - Started	0.13	.721	<.01	0.18
AOC - Recently	<0.01	.943	<.01	0.12
**Timeframe × Task Order Interaction**				
EBE Predictive	1.40	.238	.01	0.27
Causal	0.94	.334	.01	0.24
Final Predictive	1.16	.282	<.01	0.27
Continue Use	0.50	.482	<.01	0.17
Average	0.73	.395	<.01	0.21
Tally Strength	0.21	.648	<.01	0.18
AOC - Started	1.80	.181	.01	0.33
AOC - Recently	0.71	.401	<.01	0.16
**Contingency Change × Timeframe × Task Order Interaction**				
EBE Predictive	0.08	.778	<.01	0.25
Causal	0.72	.396	<.01	0.32
Final Predictive	0.02	.882	<.01	0.23
Continue Use	0.79	.375	<.01	0.28
Average	0.01	.941	<.01	0.21
Tally Strength	0.75	.388	<.01	0.34
AOC - Started	2.59	.109	.01	0.57
AOC - Recently	0.04	.837	<.01	0.22

Note: EBE Predictive = Event-by-Event Predictive. AOC = Awareness of Change.

Online Appendix 2 Table 2. Tests of Order Effects (Increasing vs. Decreasing Conditions) Within the Short Task and Within the Long Task.

	t		*p*		*Cohen's d*		*BF*		Summary	
**Measure**	**All**	**First**	**All**	**First**	**All**	**First**	**All**	**First**	**All**	**First**
**Short Timeframe**										
Causal	-0.53	-0.66	.596	.510	-.08	-0.14	.18	.27	ns	ns
Final Predictive	0.49	-1.17	.623	.246	.07	-0.24	.18	.40	ns	ns
Continue Use	-0.82	-1.01	.415	.316	-.12	-0.21	.22	.34	ns	ns
Average	-1.39	0.07	.166	.946	-.20	0.01	.39	.22	ns	ns
Tally Strength	-0.21	-0.68	.836	.497	-.03	-0.14	.16	.27	ns	ns
**Long Timeframe**										
Causal	-2.42	-2.07	.017	.041	-.35	-.42	2.27	1.38	R	R
Final Predictive	-1.61	-2.12	.110	.037	-.23	-.44	.52	1.54	ns	R
Continue Use	-2.96	-3.02	.003	.003	-.43	-.62	8.75	6.53	R	R
Average	-3.55	-3.53	<.001	<.001	-.52	-.72	44.87	40.57	R	R
Tally Strength	-2.25	-1.60	.026	.114	-.33	-.33	1.62	0.66	R	ns

Note. A positive (negative) t-value corresponds to a primacy (recency) effect. All = All data regardless of whether participants experienced the condition first or second. This corresponds to the results shown in the main paper. First = only the data from the condition that participants experienced first.

Online Appendix 2 Table 3. Tests of whether the summary judgments reveal different order effects in the short vs. long timeframe.

	F		***p***		**η_p^2**		***BF***	
**Measure**	**All**	**First**	**All**	**First**	**All**	**First**	**All**	**First**
**Main Effect of Contingency Change**								
Causal	3.90	1.03	.050	.311	.02	.01	1.00	0.27
Final Predictive	0.56	0.24	.454	.621	<.01	<.01	0.21	0.16
Continue Use	5.69	2.11	.018	.148	.03	.01	4.16	0.47
Average	9.66	6.68	.002	.011	.05	.04	45.35	3.34
Tally Strength	2.71	0.56	.102	.457	.01	<.01	0.57	0.21
**Main Effect of Timeframe**								
Causal	0.78	0.17	.379	.679	<.01	<.01	0.15	0.17
Final Predictive	0.66	0.50	.417	.479	<.01	<.01	0.13	0.19
Continue Use	0.03	<0.01	.700	.956	<.01	<.01	0.12	0.17
Average	0.28	0.29	.595	.592	<.01	<.01	0.14	0.18
Tally Strength	1.83	0.85	.177	.359	.01	<.01	0.22	0.23
**Contingency Change × Timeframe**								
Causal	2.40	3.75	.123	.054	.01	.02	0.38	1.13
Final Predictive	2.48	5.16	.117	.024	.01	.03	0.49	1.72
Continue Use	3.80	8.22	.053	.005	.02	.04	0.56	8.73
Average	3.55	6.21	.061	.014	.02	.03	0.55	3.43
Tally Strength	3.06	2.71	.082	.102	.02	.01	0.48	0.77

Note. All = All data regardless of whether participants experienced the condition first or second, so Timeframe is within subjects. The interactions in the All columns corresponds to the results in the main paper. First = only the data from the condition that participants experienced first, so Timeframe is between-subjects.
